# Second-Generation Total Synthesis of Prorocentin

**DOI:** 10.1021/acs.orglett.3c01720

**Published:** 2023-06-26

**Authors:** Kenzo Yahata, Raphael J. Zachmann, Alois Fürstner

**Affiliations:** † Max-Planck-Institut für Kohlenforschung, 45470 Mülheim/Ruhr, Germany

## Abstract

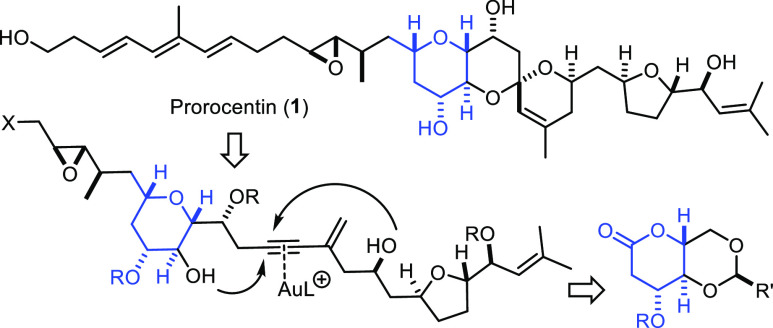

After a recent total synthesis had resolved all issues surrounding the constitution and stereostructure of prorocentin, it was possible to devise a new approach aiming at an improved supply of this scarce marine natural product; this compound is a cometabolite of the prototypical phosphatase inhibitor okadaic acid but still awaits detailed biological profiling. The revised entry starts from 2-deoxy-d-glucose; keys to success were a telescoped hemiacetal reduction/acetal cleavage and an exquisitely selective gold/Brønsted acid-cocatalyzed spiroacetalization.

Like many other dinoflagellates, the *Prorocentrum lima* strain PL021117001 collected off the Taiwanese coastline turned out to be a rich source of secondary metabolites of remarkable structural complexity. This particular microorganism was found to produce prorocentin (**1**), as well as okadaic acid (**2**) (Figure 1).^1,2^ While unmistakable structural links speak for closely coevolved biosynthesis pathways, little is currently known about a possible functional relationship between these conspicuous marine natural products. This is all the more surprising since okadaic acid is long known as a potent and selective serine/threonine phosphatase inhibitor;^3,4^ as such, **2** serves as an indispensable tool for the study of the many functions of this ubiquitous family of key regulatory enzymes. In addition, **2** is endowed with notable cytotoxic, immunotoxic, genotoxic, neurotoxic, and potentially carcinogenic properties.^3,4^ All that is known about the biological activities of prorocentin, in contrast, is its modest cytotoxicity against two human cancer cell lines and an apparent lack of antimicrobial activity against *Staphyllococcus aureus*.^1^ It is not clear at all if this seemingly rather unappealing profile reflects a disproportionally poorer intrinsic bioactivity of **1** compared with **2** or whether the compound has simply not been adequately tested to date because of the short supply. According to the literature, no more than 3 mg of prorocentin had been available for the original structure elucidation exercise and testing campaign; this amount was isolated from 450 L of a fermentation broth in which the producing *P. lima* strain had been cultured for 4 weeks at 25 °C with 8/16 h dark/light photocycles in K-nutrient-enriched seawater medium.^1^

**Figure 1:**
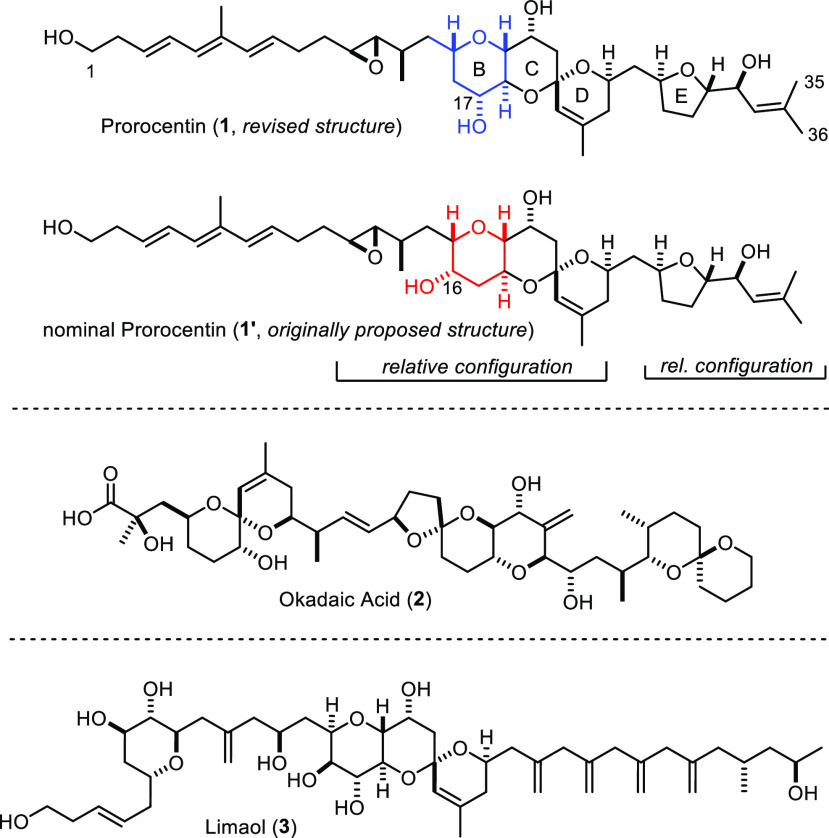
Prorocentin and related metabolites derived from dinoflagellates of the genus *Prorocentrum lima*.

We conjectured that an optimized total synthesis might provide more meaningful amounts of prorocentin, despite the intricacy of this unusual polyketide.^5^ To this end, however, it first proved necessary to clarify several subtle, yet important, structural issues. The isolation team had proposed the constitution and relative stereochemistry of the tricyclic core and the tetrahydrofuran wing, as depicted in **1**′, but had not been able to establish the stereochemical relationship between these sectors;^1^ likewise, the absolute configuration of prorocentin remained undetermined. What is more, serious doubts were raised in an early conference proceeding as to the actual structure assignment to the core;^6^ for unknown reasons, however, this claim has not been substantiated in a peer-reviewed publication in the more than 10 years that followed the preliminary disclosure.^7^

While a detailed reassessment of the published spectra certainly reinforced the doubts, an unambiguous decision as to the actual constitution of prorocentin could not be made;^8^ moreover, the data did not provide any indication whatsoever concerning the absolute configuration. Therefore, our group embarked on an extensive synthesis campaign, which targeted the originally proposed as well as a revised structure that was deemed to most likely represent the actual natural product.^8^ In doing so, we conjectured that the absolute configuration shown in Figure 1 was most likely based on the assumption that prorocentin might be a remote relative of limaol (**3**), a spirocyclic dinoflagellate-derived polyketide with confirmed stereostructure.^9−11^ In the end, this project allowed us to prove that prorocentin comprises an equatorially oriented hydroxy group at the C17 positon of the central B-ring rather than an axially disposed −OH at the adjacent C16 site, as had originally been proposed by the isolation team.^8^ The stereochemical relationship between core and wing section in **1** was established, and the absolute configuration was found to, indeed, correspond to that of limaol.

While the primary objective of this first total synthesis had been the definitive clarification of all structural issues, the acquired knowledge allowed us to shift the focus. In order to reach an improved material throughput, a shorter entry into building block **IV** representing the revised B-ring seemed necessary (Scheme 1). Originally, this sector had been derived from d-glucose in 20 steps;^8^ it was actually not so much the length of the route, which proved robust and scalable, but the fact that formation of the C15–C16 bond by ester carbonyl methylenation/olefin metathesis (**V** → **VI**)^12−14^ mandated overstoichiometric amounts of Tebbe’s reagent [Cp_2_Ti=CH_2_]^15^ generated in situ that urged us to develop an alternative approach.

**Scheme 1:**
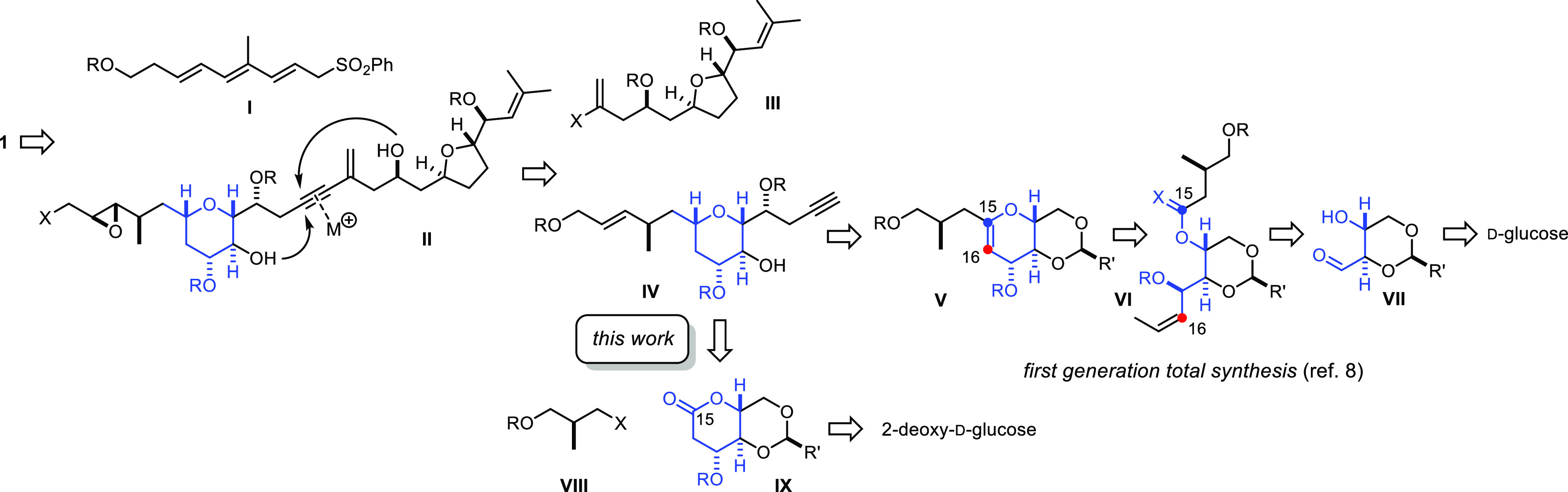
Original and Revised Retrosynthetic Analysis of Actual Prorocentin

As the reassigned B-ring structure of **1** can be mapped onto 2-deoxy-d-glucose (**4**), this sugar constituted a good starting point. Specifically, **4** was transformed on a multigram scale into naphthylidene acetal **6** under standard conditions (Scheme 2). This choice was dictated by the ease with which this particular protecting group (and the derived naphthylmethyl ethers)^16^ can be removed at a later stage without compromising any of the other substituents. Oxidation of the anomeric center with Ag_2_CO_3_/Celite^17^ was immediately followed by tert-butyldimethylsilyl (TBS) protection of the equatorially oriented C17–OH group of lactone **7** (prorocentin numbering), which proceeded in acceptable yield at scale over two steps despite the elimination-prone nature of the aldol substructure inscribed into compound **8**.

**Scheme 2:**
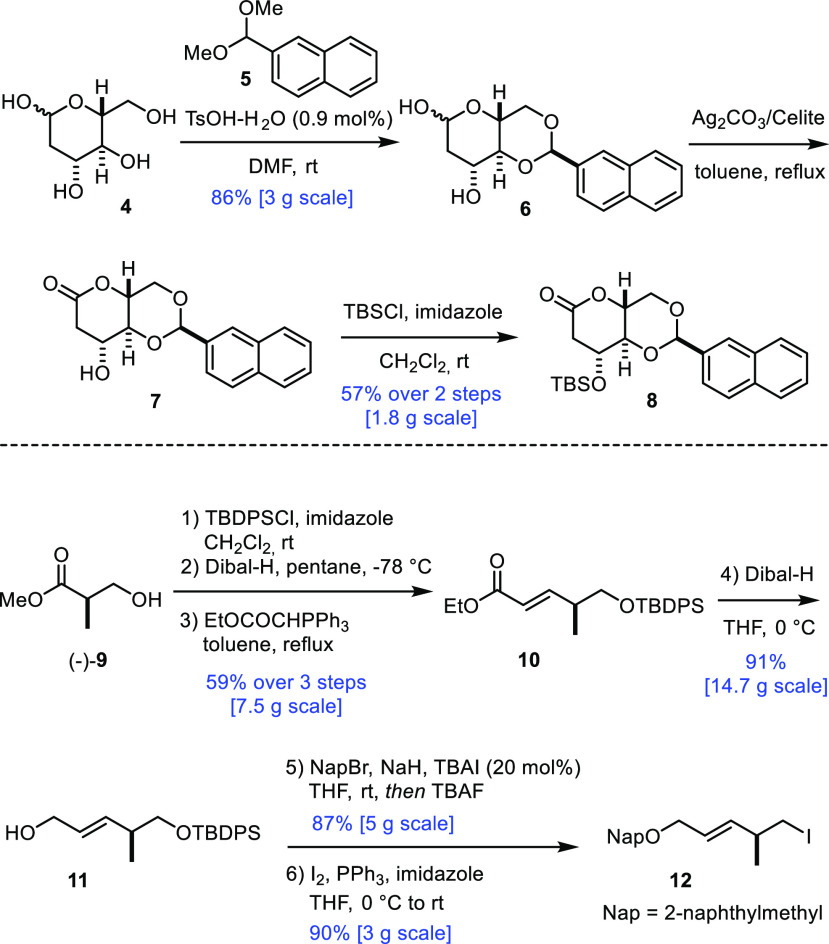
Key Building Blocks The scales shown in the Schemes refer to the amount of substrate in the single largest batch.

In parallel, Roche ester (−)-**9** was transformed into known **10** by silylation, diisobutylaluminium hydride (DIBAL-H) reduction and chain extension via Wittig olefination.^18^ Treatment of **10** with DIBAL-H in THF furnished alcohol **11**, which was protected as naphthylmethyl ether in order to remain consonant with the regimen chosen for the sugar fragment.^16^ The other terminus was then readily elaborated into primary iodide **12**.

Coupling of iodide **12** with lactone **8** was achieved upon slow addition of *tert*-BuLi to a solution of the two reaction partners in THF at −78 °C; under these Barbier conditions, the organolithium reagent derived from **12** by metal/halogen exchange was effectively trapped by admixed **8** to give product **13** in high yield (Scheme 3). As the reduction of a lactol to the corresponding *C*-glycoside^19^ and the projected regioselective cleavage of the benzylidene-type acetal are both typically achieved by a Lewis acid promotor in combination with an appropriate hydride source, we saw the opportunity for a telescoped approach. After some experimentation (for details, see the Supporting Information), it was found that treatment of a solution of **13** in CH_2_Cl_2_ with TBSOTf and excess Et_3_SiH at −78 °C in the presence of 4 Å molecular sieves (MS) led to the stereoselective reduction of the anomeric −OH group;^20^ subsequent addition of PhBCl_2_ at the same low temperature then entailed selective cleavage of the acetal ring to give alcohol **14** as the only detectable isomer.^21−23^ As expected, the side chain in **14** branching off the tetrahydropyran ring is equatorially disposed, and the primary −OH group has been exclusively unveiled. Importantly, this involved process scaled well.

**Scheme 3:**
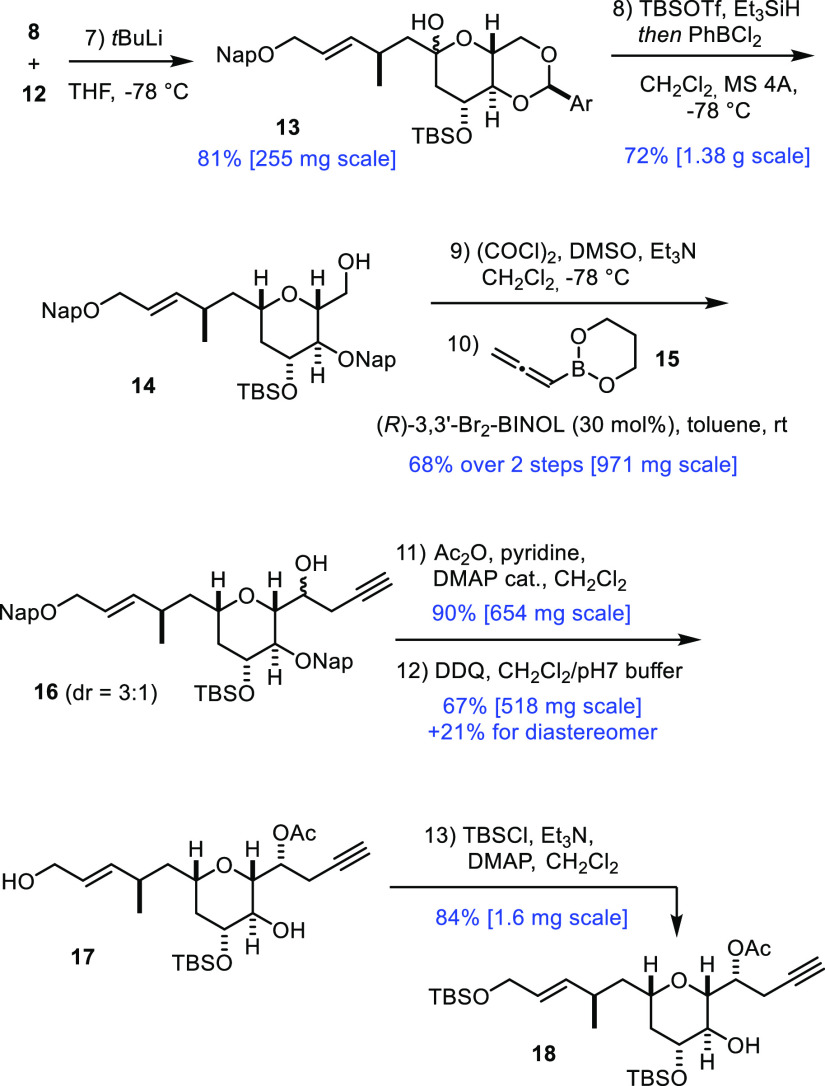
New Approach to the Central B-Ring Fragment

Oxidation of the primary alcohol in **14** followed by diastereoselective addition of allenylboronate **15** to the resulting aldehyde catalyzed by (*R*)-3,3′-dibromo-Binol furnished homopropargyl alcohol **16** in good yield.^24^ Although the diastereoselectivity (dr ≈ 3:1) was lower than that observed in the analogous transformation of our first-generation approach,^8,25^ the scalability and preparative convenience of this organocatalytic step were compelling. Acetylation followed by cleavage of both naphthylmethyl ethers by 4,5-dichloro-3,6-dioxo-1,4-cyclohexadiene-1,2-dicarbonitrile (DDQ) gave diol **17**; it was at this stage that the diastereomers formed in the propargylation step could be separated by flash chromatography.

An aliquot of compound **17** was selectively silylated at the primary allylic −OH group in order to intercept our previous route; comparison proved the identity of the samples and, hence, confirmed the stereochemical assignment.^8^ Product **18** had previously been formed in 20 steps from d-glucose along the longest linear sequence, whereas the new route (13 steps) is considerably shorter.

On the way to the natural product, however, the TBS–ether formation is unnecessary, and an additional step can, therefore, be saved (Scheme 4). Thus, alkyne **17** was engaged into a high-yielding Sonogashira reaction,^26^ with alkenyl iodide **19** representing the “eastern” sector of prorocentin,^8^ which set the stage for the critical gold-catalyzed spirocyclization reaction.^27−31^ In line with our expectations,^8,9,32,33^ this key transformation worked exquisitely well when cocatalyzed by [(JohnPhos)Au(MeCN)]SbF_6_ (**21**, 10 mol %) and pyridinium *p*-toluenesulfonate (PPTS, 10 mol %) in CH_2_Cl_2_; within the limits of detection (^1^H NMR), compound **24** was the only isomer present in the crude mixture. The gold complex is thought to trigger the decisive first 6-*endo*-dig cyclization, which ultimately translates into the 6/6-spirocyclic array (**20** → **22**), whereas the Brønsted acid cocatalyst accounts for the fully regioselective isomerization of the *exo*-methylene group to the endocyclic position, likely at the stage of the transient oxocarbenium intermediate **23**; at the same time, the reversibility of the acid-catalyzed step ensures thermodynamic control over the configuration of the doubly anomeric spiroacetal center.^34−36^

**Scheme 4:**
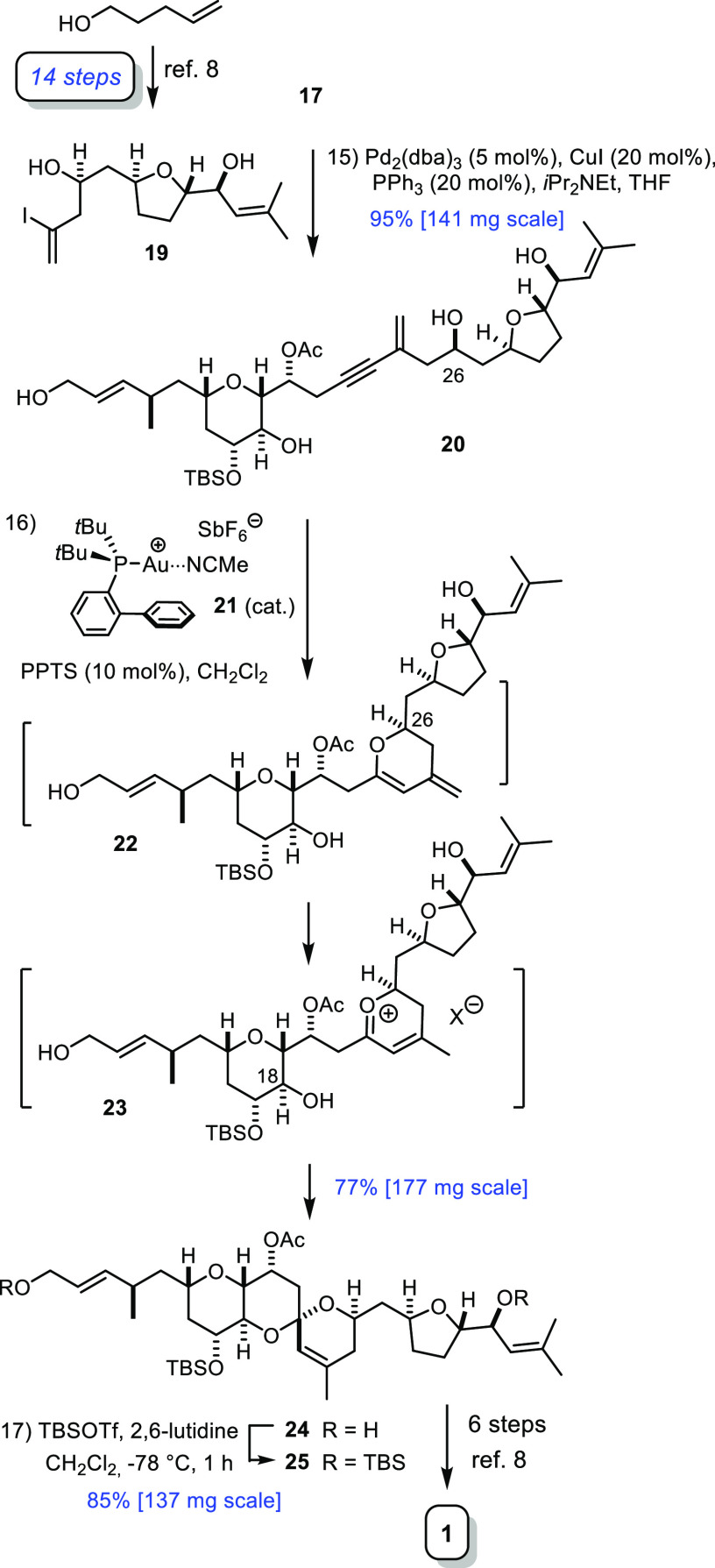
Completion of the Total Synthesis

Disilylation of **24** with formation of **25** intercepted our original route to prorocentin;^8^ the step-count is favorable (17 versus 23 steps),^37^ a good material throughput is secured, and the technically challenging ester carbonyl/olefin metathesis reaction mediated by [Cp_2_Ti=CH_2_] is altogether avoided. An aliquot of **25** was then elaborated into prorocentin (**1**) by following the established route.^8^ In consideration of the unknown risks exerted by this intricate marine natural product, the run was deliberately limited to 35 mg of the final compound, but we see no reason why the new route could not be scaled up to a significant extent if necessary. In any case, the now available amount should allow for more detailed biological profiling; pertinent results will be reported in due time.

## Data Availability

The data underlying this study are available in the published article and its Supporting Information.

## Supporting Information Available

Detailed experimental procedures, analytical and spectroscopic data, and copies of NMR spectra of new compounds (PDF)
