# Supplementary material for: Second-Generation Total Synthesis of Prorocentin
Source: Org Lett. 2023 Jun 26;25(26):4903–7. doi: 10.1021/acs.orglett.3c01720 (PMC10334468; doi:10.1021/acs.orglett.3c01720)
Supplement: Supplementary file 1 — ol3c01720_si_001.pdf [file ol3c01720_si_001.pdf]

# SUPPORTING INFORMATION

## Second-Generation Total Synthesis of Prorocentin

Kenzo Yahata, Raphael J. Zachmann, Alois Fürstner*

*Max-Planck-Institut für Kohlenforschung, 45470 Mülheim/Ruhr, Germany*

Email: fuerstner@kofo.mpg.de

**TABLE OF CONTENTS**

GENERAL INFORMATION .......... S2

CENTRAL FRAGMENT .......... S3

COPIES OF SPECTRA .......... S13

REFERENCES .......... S28

## GENERAL INFORMATION

Unless stated otherwise, all reactions were carried out under argon in preiously flame-dried glassware, ensuring rigorously inert conditions. The solvents were purified by distillation over the indicated drying agents and were stored and handled under argon: THF, $Et_2O$ (Mg/anthracene); hexanes, toluene (Na/K); $NEt_3$, diisopropylamine, diisopropylethylamine, 2,6-lutidine, pyridine, *tert*-butyl methyl ether, $CH_2Cl_2$, DMPU ($CaH_2$); MeOH (Mg, stored over 3Å MS); DMSO, DMF, 1,4-dioxane, and $CH_3CN$ were dried by an adsorption solvent purification system based on molecular sieves.

Thin layer chromatography (TLC): Macherey-Nagel precoated plates (POLYGRAM®SIL/UV254); flash chromatography: Merck silica gel 60 (40-63 µm or 15-40 µm - referred to as “fine silica”) with pre-distilled or HPLC grade solvents.

NMR spectra were recorded on Bruker AV III 400, AV III 600 or AV NEO 600 spectrometers in the solvents indicated; chemical shifts (δ) are given in ppm relative to TMS, coupling constants (*J*) in Hz. The solvent signals were used as references and the chemical shifts converted to the TMS scale ($CDCl_3$: $\delta_C$ = 77.16 ppm; residual $CHCl_3$: $\delta_H$ = 7.26 ppm; $CD_2Cl_2$: $\delta_C$ = 53.84 ppm; residual $CHDCl_2$: $\delta_H$ = 5.32 ppm; $C_6D_6$: $\delta_C$ = 128.06 ppm; residual $C_6HD_5$: $\delta_H$ = 7.16 ppm; $CD_3OD$: $\delta_C$ = 49.00 ppm; residual $CHD_2OD$: $\delta_H$ = 3.31 ppm; $D_3C(C{=}O)CD_3$: $\delta_C$ = 29.84 ppm; residual $D_3C(C{=}O)CHD_2$: $\delta_H$ = 2.05 ppm).

IR: Alpha Platinum ATR (Bruker), wavenumbers ($\tilde{\nu}$) in $cm^{-1}$.

MS (EI): Finnigan MAT 8200 (70 eV), DI-MS (EI): Finnigan MAT SSQ 7000, ESI-MS: ESQ 3000 (Bruker) or Thermo Scientific LTQ-FT or Thermo Scientific Exactive. HRMS: Bruker APEX III FT-MS (7 T magnet) or MAT 95 (Finnigan) or Thermo Scientific LTQ-FT or Thermo Scientific Exactive. GC-MS was measured on a Shimadzu GCMS-QP2010 Ultra instrument.

Unless stated otherwise, all commercially available compounds (abcr, Acros, TCI, Aldrich, Alfa Aesar) were used without further purification.

Light-sensitive reactions were carried out in glassware wrapped in aluminum foil with the fume hood light turned off.

## CENTRAL FRAGMENT

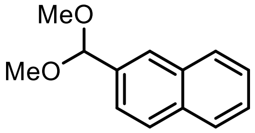


**2-(Dimethoxymethyl)naphthalene (5).** Trimethyl orthoformate (10.5 mL, 96.0 mmol) and *p*-toluenesulfonic acid monohydrate (2.44 g, 12.8 mmol) were added to a solution of 2-naphthaldehyde (10.0 g, 64.0 mmol) in methanol (100 mL). The mixture was stirred for 16 h at room temperature. The solvent was removed under reduced pressure and the residue was purified by flash chromatography (silica, hexanes/EtOAc 20:1) to provide the title compound as a colorless oil (12.8 g, 99%). $^1$H NMR (400 MHz, $C_6D_6$) δ 8.07 (s, 1H), 7.74 – 7.58 (m, 4H), 7.29 – 7.21 (m, 2H), 5.48 (s, 1H), 3.17 (s, 6H). The spectral data matched the literature.[1]

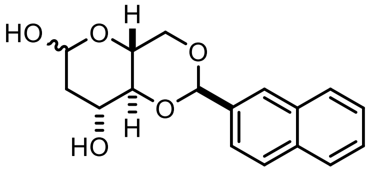


**Lactol 6.** 2-(Dimethoxymethyl)naphthalene **5** (4.40 g, 21.9 mmol) and *p*-toluenesulfonic acid monohydrate (30.0 mg, 158 µmol) were added to a solution of 2-deoxy-D-glucose (**4**, 3.00 g, 18.3 mmol) in DMF (18 mL). After stirring for 15 h at room temperature, the reaction was quenched with $Et_3N$ (0.1 mL) and the resulting mixture was concentrated under reduced pressure. The residue was purified by flash chromatography (silica, hexanes/EtOAc 1:1 → 1:2) to provide the title compound as an inseparable mixture of diastereoisomers in the form of a colorless amorphous solid (dr ≈ 3:2, 4.75 g, 86%). $[\alpha]_{20}^{D}$ = +30.4 (c = 0.50, $CH_2Cl_2$). $^1$H NMR (400 MHz, MeOD) δ 8.04 – 7.97 (m, 1H), 7.92 – 7.78 (m, 3H), 7.66 – 7.58 (m, 1H), 7.51 – 7.42 (m, 2H), 5.76 (s, 0.6H), 5.74 (s, 0.4H), 5.30 (dd, *J* = 3.7, 1.1 Hz, 0.6H), 4.91 (dd, *J* = 9.8, 2.3 Hz, 0.4H), 4.28 (dd, *J* = 10.3, 4.7 Hz, 0.4H), 4.24 – 4.12 (m, 1.2H), 4.02 (td, *J* = 9.9, 4.9 Hz, 0.6H), 3.92 – 3.86 (m, 0.4H), 3.86 – 3.75 (m, 1H), 3.56 – 3.35 (m, 1.4H), 2.25 (ddd, *J* = 12.8, 5.1, 2.2 Hz, 0.4H), 2.15 (ddd, *J* = 13.2, 5.2, 1.3 Hz, 0.6H), 1.75 (ddd, *J* = 13.2, 11.3, 3.7 Hz, 0.6H), 1.60 (ddd, *J* = 12.8, 11.4, 9.8 Hz, 0.4H). $^{13}$C NMR (101 MHz, MeOD) δ 136.7, 136.6, 135.0, 134.31, 134.29, 129.31, 129.29, 128.73, 128.67, 127.43, 127.41, 127.18, 127.16, 126.8, 125.22, 125.18, 103.1, 103.0, 95.7, 93.5, 85.5, 84.6, 70.3, 69.9, 69.0, 68.0, 66.4, 64.2, 42.3, 40.0. IR (film) ṽ 3411, 2936, 2868, 1377, 1346, 1268, 1176, 1125, 1092, 1026, 989, 925, 860, 823, 747, 478 $cm^{-1}$. HRMS (ESI$^+$) Calcd for $C_{17}H_{18}NaO_5$ $[M+Na]^+$: 325.1046. Found: 325.1049.

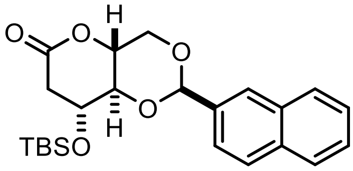


**Lactone 8.** $Ag_2CO_3$ on Celite (50% *w/w*, 6.60 g, 11.9 mmol) was added to a suspension of lactol **6** (1.80 g, 5.95 mmol) in toluene (50 mL) at 80 °C in the dark. After stirring for 1 h at reflux temperature (oil bath) in the dark, the mixture was cooled to room temperature, diluted with acetone (50 mL), and filtered through a pad of Celite, which was thoroughly rinsed with acetone. The combined filtrates were concentrated under reduced pressure to give the crude lactone **7** as a yellow solid, which was subjected to the next step without further purification.

Imidazole (2.40 g, 35.7 mmol) and TBSCl (2.70 g, 17.9 mmol) were added at room temperature to a solution of the crude lactone **7** in $CH_2Cl_2$ (90 mL). After stirring for 15 h, the reaction was quenched with $H_2O$ (20 mL), the phases were separated and the aqueous layer was extracted with $CH_2Cl_2$ (2 × 20 mL). The combined organic layers were dried over $Na_2SO_4$, filtered, and concentrated under reduced pressure. The residue was purified by flash chromatography (silica, hexanes/*tert*-butyl methyl ether 6:1 → 3:1) to provide the title compound as a white amorphous solid material (1.41 g, 57% over two steps). $[\alpha]_{20}^{D}$ = –30.0 (c = 1.00, $CH_2Cl_2$). $^1$H NMR (400 MHz, $CD_2Cl_2$) δ 8.03 – 7.97 (m, 1H), 7.93 – 7.83 (m, 3H), 7.60 (dd, *J* = 8.6, 1.7 Hz, 1H), 7.55 – 7.49 (m, 2H), 5.76 (s, 1H), 4.50 (dd, *J* = 10.5, 5.2 Hz, 1H), 4.30 (td, *J* = 7.9, 5.4 Hz, 1H), 4.14 (td, *J* = 10.1, 5.2 Hz, 1H), 3.89 (t, *J* = 10.3 Hz, 1H), 3.81 (dd, *J* = 9.9, 7.7 Hz, 1H), 3.16 (dd, *J* = 17.7, 8.0 Hz, 1H), 2.64 (dd, *J* = 17.9, 5.6 Hz, 1H), 0.91 (s, 9H), 0.11 (s, 3H), 0.10 (s, 3H). $^{13}$C NMR (101 MHz, $CD_2Cl_2$) δ 168.9, 134.9, 134.0, 133.2, 128.7, 128.3, 128.0, 127.0, 126.7, 125.9, 124.1, 102.0, 81.8, 68.7, 68.4, 68.1, 39.8, 25.8, 18.4, -4.4, -4.8. IR (film) ṽ 2953, 2929, 2857, 1757, 1471, 1378, 1256, 1228, 1176, 1128, 1101, 1080, 945, 890, 838, 780, 477 cm$^{-1}$. HRMS (ESI$^+$) Calcd for $C_{23}H_{30}NaO_5Si$ [M+Na]$^+$: 437.1754. Found: 437.1753.

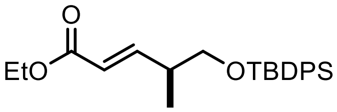


**Ester 10.** Imidazole (8.60 g, 126 mmol) and TBDPSCl (19.7 mL, 75.8 mmol) were added to a solution of methyl (*R*)-(−)-3-hydroxy-2-methylpropionate ((−)-**9**, 7.00 mL, 63.2 mmol) in $CH_2Cl_2$ (120 mL) at room temperature. After stirring for 2 h, the mixture was diluted with hexanes (200 mL) and washed with $H_2O$ (2 × 50 mL). The organic phase was dried over $Na_2SO_4$ and the solvent removed under reduced pressure. The obtained crude TBDPS ether was used in the next step without further purification.

To a solution of the crude TBDPS ether in pentane (400 mL) was added a solution of Dibal-H (1 M in hexane, 70.0 mL, 70.0 mmol) at –78 °C. After stirring for 30 min, the reaction was quenched upon careful addition of acetone (5 mL) followed by sat. aq. Rochelle’s salt solution (200 mL). The resulting mixture was vigorously stirred for 2 h at room temperature until clean phase separation was reached. The aqueous layer was extracted with $CH_2Cl_2$ (2 × 100 mL) and the combined organic layers were dried over $Na_2SO_4$, filtered, and concentrated under reduced pressure. The crude aldehyde was used in the next step without further purification.

(Ethoxycarbonylmethylene)-triphenylphosphorane (44.0 g, 126 mmol) was added to a solution of the crude aldehyde in toluene (300 mL) and the resulting suspension was stirred at reflux temperature (oil bath) for 5 h. After cooling to room temperature, the solvent was removed under reduced pressure and the residue was purified by flash chromatography (silica, hexanes/EtOAc 20:1) to provide the title compound as a colorless oil (14.7 g, 59% over three steps). $^1$H NMR (400 MHz, $CDCl_3$) δ 7.66 (dq, *J* = 6.5, 1.4 Hz, 4H), 7.48 – 7.33 (m, 6H), 6.97 (dd, *J* = 15.8, 7.2 Hz, 1H), 5.84 (dd, *J* = 15.7, 1.4 Hz, 1H), 4.20

(q, $J$ = 7.2 Hz, 2H), 3.65 – 3.52 (m, 2H), 2.62 – 2.51 (m, 1H), 1.30 (t, $J$ = 7.2 Hz, 3H), 1.08 (d, $J$ = 6.7 Hz, 3H), 1.06 (s, 9H). The spectral data matched the literature.[2]

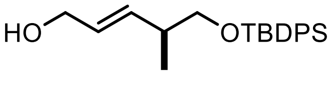


**Alcohol 11.** Dibal-H (1 M in THF, 95 mL, 95 mmol) was added to a solution of ester **10** (14.7 g, 37.1 mmol) in THF (200 mL) at 0 °C and stirring was continued for 1 h. EtOAc (100 mL) and sat. aq. Rochelle´s salt solution (200 mL) were added and the mixture was stirred vigorously for 1 h. The layers were separated and the aqueous phase was extracted with EtOAc (3 × 100 mL). The combined organic layers were washed with brine and dried over $Na_2SO_4$. The solvent was removed under reduced pressure and the residue purified by flash chromatography (silica, hexanes/*tert*-butyl methyl ether 3:1 → 12:5) to provide the title compound as a colorless oil (12.0 g, 91%). $[\alpha]_D^{20}$ = –6.8 (c = 1.00, $CH_2Cl_2$). $^1$H NMR (400 MHz, $CD_2Cl_2$) δ 7.72 – 7.61 (m, 4H), 7.47 – 7.32 (m, 6H), 5.71 – 5.55 (m, 2H), 4.03 (d, $J$ = 4.0 Hz, 2H), 3.61 – 3.47 (m, 2H), 2.52 – 2.32 (m, 1H), 1.04 (m, 12H). $^{13}$C NMR (101 MHz, $CD_2Cl_2$) δ 136.0, 135.3, 134.4, 130.0, 129.5, 128.0, 69.0, 64.0, 39.4, 27.0, 19.6, 16.7. IR (film) ṽ 3323, 2931, 2857, 1427, 1110, 1084, 1007, 971, 824, 740, 701, 614, 505, 489 cm$^{-1}$. HRMS (ESI$^+$) Calcd for $C_{22}H_{30}O_2SiNa$ [M+Na]$^+$: 377.1907. Found: 377.1908.

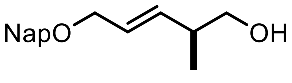


**Naphthyl methyl ether S1.** Alcohol **11** (5.00 g, 14.1 mmol) was slowly added to a suspension of NaH (423 mg, 17.6 mmol) in THF (30 mL) at 0 °C. The mixture was then stirred at room temperature for 90 min. TBAI (1.04 g, 2.82 mmol) and 2-(bromomethyl)naphthalene (3.90 g, 17.6 mmol) were introduced and stirring was continued overnight. TBAF•3$H_2O$ (9.22 g, 29.2 mmol) was added and stirring was continued for 8 h before a solution of TBAF (1 M in THF, 15.0 mL, 15.0 mmol) was added. After stirring overnight, sat. aq. $NH_4Cl$ (50 mL) was added and the mixture was extracted with *tert*-butyl methyl ether (3 × 50 mL). The combined organic layers were washed with brine and dried over $Na_2SO_4$. The solvent was removed under reduced pressure and the residue was purified by flash chromatography (silica, hexanes/*tert*-butyl methyl ether 1:1) to provide the title compound as a colorless oil (3.13 g, 87%). $[\alpha]_D^{20}$ = –20.1 (c = 1.00, $CH_2Cl_2$). $^1$H NMR (400 MHz, $CD_2Cl_2$) δ 7.89 – 7.76 (m, 4H), 7.53 – 7.42 (m, 3H), 5.77 – 5.58 (m, 2H), 4.65 (d, $J$ = 0.9 Hz, 2H), 4.05 (dd, $J$ = 5.5, 1.0 Hz, 2H), 3.53 – 3.37 (m, 2H), 2.45 – 2.30 (m, 1H), 1.47 – 1.39 (m, 1H), 1.02 (d, $J$ = 6.9 Hz, 3H). $^{13}$C NMR (101 MHz, $CD_2Cl_2$) δ 136.7, 136.6, 133.7, 133.3, 128.3, 128.2, 128.0, 127.9, 126.6, 126.4, 126.3, 126.2, 72.5, 71.2, 67.5, 39.8, 16.5. IR (film) ṽ 3400, 2957, 2927, 2867, 1366, 1124, 1092, 1073, 1036, 972, 855, 817, 752, 475 cm$^{-1}$. HRMS (ESI$^+$) Calcd for $C_{17}H_{20}O_2Na$ [M+Na]$^+$: 279.1355. Found: 279.1356.

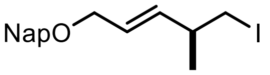


**Iodide 12**. Imidazole (2.43 g, 35.7 mmol) and $PPh_3$ (4.68 g, 17.8 mmol) were added to a solution of naphthyl methyl ether **S1** (3.05 g, 11.9 mmol) in THF

(60 mL). The mixture was cooled to 0 °C before $I_2$ (3.62 g, 14.3 mmol) was added and stirring was continued at this temperature for 30 min and for another 90 min at ambient temperature. *tert*-Butyl methyl ether (100 mL) was introduced, and the mixture was washed with sat. aq. $Na_2S_2O_3$ (100 mL), water (100 mL) and brine (100 mL). The organic phase was evaported and the residue purified by flash chromatography (silica, hexanes/toluene 4:1 $\rightarrow$ hexanes/*tert*-butyl methyl ether 20:1) to provide the title compound as a colorless oil (3.91 g, 90%). $[\alpha]_D^{20}$ = –16.5 (c = 1.00, $CH_2Cl_2$). $^1$H NMR (400 MHz, $CD_2Cl_2$) δ 7.89 – 7.78 (m, 4H), 7.53 – 7.43 (m, 3H), 5.74 – 5.57 (m, 2H), 4.67 (d, *J* = 0.8 Hz, 2H), 4.08 – 4.02 (m, 2H), 3.26 – 3.14 (m, 2H), 2.42 (hept, *J* = 6.5 Hz, 1H), 1.14 (d, *J* = 6.7 Hz, 3H). $^{13}$C NMR (101 MHz, $CD_2Cl_2$) δ 137.0, 136.6, 133.7, 133.3, 128.3, 128.2, 128.0, 127.7, 126.7, 126.4, 126.3, 126.2, 72.3, 70.9, 38.8, 20.7, 15.4. IR (film) ṽ 2964, 2850, 1367, 1195, 1169, 1124, 1100, 969, 854, 816, 751, 475 cm$^{-1}$. HRMS (GC-EI) Calcd for $C_{17}H_{19}OI$ $[M]^+$: 366.0475. Found: 366.0474.

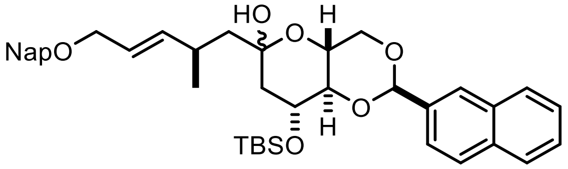


**Hemiketal 13.** *tert*-BuLi (1.7 M in pentane, 840 µL, 1.25 mmol) was added in three portions over the course of 60 min to a solution of lactone **8** (255 mg, 615 µmol) and iodide **12** (450 mg, 1.23 mmol) in THF (22 mL) at –78 °C. After stirring for additional 30 min, the reaction was quenched upon careful addition of sat. aq. $NH_4Cl$ (20 mL). The layers were separated and the aqueous layer was extracted with *tert*-butyl methyl ether (3 × 20 mL). The combined organic phases were dried over $Na_2SO_4$, the solvent was removed under reduced pressure, and the residue was purified by flash chromatography (silica, hexanes/*tert*-butyl methyl ether 4:1 $\rightarrow$ 3:1) to provide the title compound as a colorless oil (mixture of anomers, 325 mg, 81%). $[\alpha]_{20}^{D}$ = –23.9 (c = 1.00, $CH_2Cl_2$). $^1$H NMR (400 MHz, $CD_2Cl_2$) δ 8.01 – 7.76 (m, 16H), 7.65 – 7.42 (m, 12H), 5.85 – 5.51 (m, 6H), 4.72 – 4.66 (m, 3H), 4.62 (s, 2H), 4.33 (dd, *J* = 10.7, 5.3 Hz, 1H), 4.29 – 4.17 (m, 2H), 4.10 – 3.93 (m, 6H), 3.81 – 3.72 (m, 2H), 3.64 (t, *J* = 10.4 Hz, 1H), 3.46 (t, *J* = 9.3 Hz, 1H), 3.22 (d, *J* = 2.3 Hz, 1H), 2.94 (dd, *J* = 16.6, 4.7 Hz, 1H), 2.88 (d, *J* = 2.8 Hz, 1H), 2.81 – 2.65 (m, 3H), 2.50 (dd, *J* = 16.6, 6.6 Hz, 1H), 2.42 (dd, *J* = 16.7, 7.2 Hz, 1H), 2.17 (dd, *J* = 13.1, 5.2 Hz, 1H), 1.79 – 1.52 (m, 3H), 1.06 (d, *J* = 6.8 Hz, 3H), 0.97 (d, *J* = 6.7 Hz, 3H), 0.91 (s, 9H), 0.90 (s, 9H), 0.21 (s, 3H), 0.12 (s, 3H), 0.10 (s, 3H), 0.07 (s, 3H). $^{13}$C NMR (101 MHz, $CD_2Cl_2$) δ 207.5, 141.1, 138.6, 136.7, 136.6, 136.0, 135.6, 134.0, 133.9, 133.7, 133.4, 133.34, 133.30, 133.26, 128.70, 128.66, 128.4, 128.34, 128.29, 128.22, 128.20, 128.1, 128.02, 127.99, 126.9, 126.8, 126.7, 126.64, 126.57, 126.5, 126.4, 126.3, 126.21, 126.19, 125.9, 125.8, 125.6, 124.5, 124.3, 101.9, 101.6, 99.6, 84.6, 81.5, 72.6, 72.3, 71.3, 71.2, 70.9, 70.1, 69.8, 67.9, 64.4, 63.2, 51.2, 48.4, 46.2, 44.7, 32.6, 32.1, 26.0, 22.6, 20.1, 18.5, 18.3, -4.3, -4.6, -4.66, -4.68. IR (film) ṽ 3515, 2954, 2928, 2855, 1756, 1715, 1470, 1376, 1253, 1174, 1126, 1096, 857, 837, 818, 780, 747, 476 cm$^{-1}$. HRMS (ESI$^+$) Calcd for $C_{40}H_{50}NaO_6Si$ $[M+Na]^+$: 677.3268. Found: 677.3272.

**Telescoped Lactol Reduction/Acetal Cleavage**

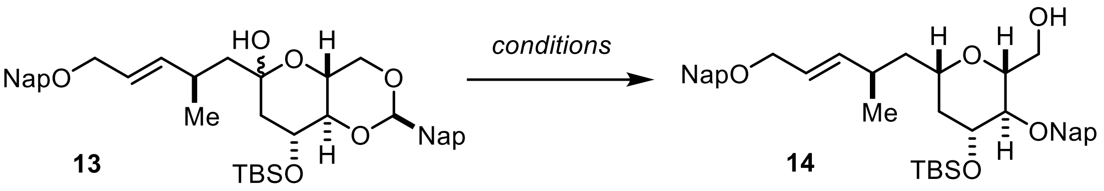


| Entry | Conditions | Yield |
|---|---|---|
| 1 | 1. Step: TMSOTf (1.2 equiv.), $Et_3SiH$ (5 equiv.), $CH_2Cl_2$, –78°C;<br>2. Step: Dibal-H (4 equiv.), $CH_2Cl_2$, –78°C → –40°C | (1) 56% / (2) 74% |
| 2 | $PhBCl_2$ (3 equiv.), $Et_3SiH$ (5 equiv.), $CH_2Cl_2$, –78°C | 16% |
| 3 | $PhBCl_2$ (3 equiv.), $Et_3SiH$ (5 equiv.), $Et_2O$, –78°C → –30°C | 50% |
| 4 | TBSOTf (4 equiv.), $Et_3SiH$ (8 equiv.), MS 4Å, $CH_2Cl_2$, –78°C; *then* $PhBCl_2$ (3.5 equiv.), –78°C | 56% |
| 5 | TBSOTf (2 equiv.), $Et_3SiH$ (8 equiv.), MS 4Å, $CH_2Cl_2$, –78°C; *then* $PhBCl_2$ (3.5 equiv.), –78°C | 76% (54 mg scale)<br>72% (1.38 g scale) |

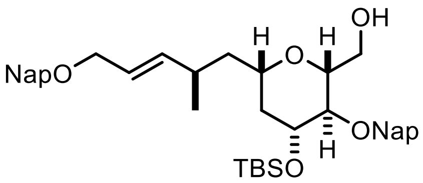


**Alcohol 14.** Molecular sieves 4Å (2.00 g) and $Et_3SiH$ (2.70 mL, 16.9 mmol) were added to a solution of hemiketal **13** (1.38 g, 2.11 mmol) in $CH_2Cl_2$ (46 mL). After stirring for 1 h, TBSOTf (970 µL, 4.22 mmol) was introduced at –78 °C and stirring continued for 2 h at this temperature before a solution of $PhBCl_2$ (950 µL, 7.32 mmol) in $CH_2Cl_2$ (5 mL) was added dropwise. The resulting mixture was stirred for another 40 min at –78°C before a solution of $Et_3N$ (5 mL) and MeOH (5 mL) in *tert*-butyl methyl ether (20 mL) was added. After warming to ambient temperature, sat. aq. $NaHCO_3$ (40 mL) was introduced and the phases were separated. The aqueous layer was extracted with *tert*-butyl methyl ether (3 × 20 mL) and the combined extracts were dried over $Na_2SO_4$. The solvent was removed under reduced pressure and the residue was purified by flash chromatography (silica, hexanes/*tert*-butyl methyl ether 3:1) to provide the title compound as a colorless oil (975 mg, 72%). $[\alpha]_{20}^{D}$ = –18.8 (c = 1.00, $CH_2Cl_2$). $^1H$ NMR (400 MHz, $C_6D_6$) δ 7.86 – 7.82 (m, 1H), 7.76 (dd, *J* = 1.6, 0.8 Hz, 1H), 7.73 – 7.57 (m, 6H), 7.46 (ddd, *J* = 9.4, 8.4, 1.6 Hz, 2H), 7.31 – 7.19 (m, 4H), 5.67 (dtd, *J* = 15.5, 5.8, 0.8 Hz, 1H), 5.52 (ddt, *J* = 15.5, 8.2, 1.3 Hz, 1H), 5.10 (dd, *J* = 11.9, 0.9 Hz, 1H), 4.82 (dd, *J* = 11.8, 1.0 Hz, 1H), 4.54 (s, 2H), 3.98 – 3.87 (m, 3H), 3.87 – 3.69 (m, 2H), 3.54 – 3.33 (m, 2H), 3.27 (ddd, *J* = 9.6, 4.7, 2.7 Hz, 1H), 2.52 – 2.40 (m, 1H), 2.04 (s, 1H), 1.78 (ddd, *J* = 12.9, 5.3, 2.0 Hz, 1H), 1.56 – 1.43 (m, 2H), 1.14 (ddd, *J* = 13.8, 10.1, 3.3 Hz, 1H), 1.00 (s, 9H), 0.96 (d, *J* = 6.7 Hz, 3H),

0.10 (s, 3H), 0.05 (s, 3H). $^{13}$C NMR (101 MHz, $CD_2Cl_2$) δ 139.1, 136.4, 136.3, 133.33, 133.29, 132.93, 132.88, 128.0, 127.9, 127.84, 127.80, 127.6, 126.21, 126.17, 126.1, 126.0, 125.86, 125.86, 125.8, 125.6, 80.3, 78.9, 74.8, 74.4, 73.2, 71.9, 70.9, 62.8, 42.5, 41.4, 33.3, 25.7, 20.9, 17.8, -4.5, -4.7. IR (film) ṽ 3479, 2952, 2927, 2855, 1461, 1373, 1253, 1092, 973, 852, 837, 815, 776, 753, 475 cm$^{-1}$. HRMS (ESI$^+$) Calcd for $C_{40}H_{52}NaO_5Si$ [M+Na]$^+$: 663.3476. Found: 663.3478.

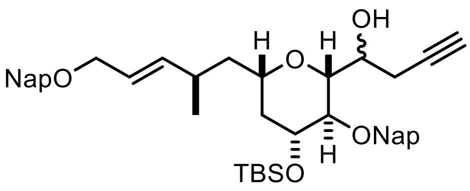


**Homopropargylic alcohol 16.** A solution of DMSO (540 µL, 7.60 mmol) in $CH_2Cl_2$ (5 mL) was added at –78 °C to a solution of oxalyl chloride (260 µL, 3.03 mmol) in $CH_2Cl_2$ (10 mL). After stirring for 15 min, a solution of alcohol **14** (971 mg, 1.52 mmol) in $CH_2Cl_2$ (15 mL) was added, and the resulting mixture was stirred at this temperature for 30 min before $Et_3N$ (1.10 mL, 7.89 mmol) was introduced. After warming to 0 °C and stirring for additional 15 min, the reaction was quenched with sat. aq. $NH_4Cl$ (20 mL). The aqueous layer was extracted with *tert*-butyl methyl ether (3 × 20 mL) and the combined organic phases were washed with brine and dried over $Na_2SO_4$. Toluene (10 mL) was added, before the more volatile solvents were removed under reduced pressure to provide a solution of the crude aldehyde in toluene, which was directly used in the next step without further purification.

Toluene (14 mL), (*R*)-(+)-3,3′-dibromo-1,1′-bi-2-naphthol (200 mg, 450 µmol), and allenyl boronate **15** (300 mg, 2.42 mmol) were added to a solution of the crude aldehyde in toluene (10 mL). After stirring for 14 h, the solvent was removed under reduced pressure and the residue was purified by flash chromatography (silica, hexanes/*tert*-butyl methyl ether 6:1 → 4:1) to provide the title compound as an inseparable mixture of diastereomers in form of a colorless oil (dr ≈ 3:1, 704 mg, 68% over two steps). $^1$H NMR (400 MHz, $CD_2Cl_2$) δ 7.91 – 7.77 (m, 8H), 7.55 – 7.42 (m, 6H), 5.73 – 5.50 (m, 2H), 5.23 (major isomer, dd, *J* = 11.7, 0.8 Hz, 0.75H), 5.11 (minor isomer, d, *J* = 12.3 Hz, 0.25H), 4.90 – 4.81 (m, 1H), 4.67 (s, 2H), 4.09 – 3.84 (m, 4H), 3.53 – 3.21 (m, 3H), 2.60 – 2.40 (m, 3H), 2.02 (minor isomer, t, *J* = 2.7 Hz, 0.25H), 1.97 (major isomer, t, *J* = 2.7 Hz, 0.75H), 1.93 – 1.82 (m, 1H), 1.65 – 1.24 (m, 3H), 1.07 – 1.02 (m, 3H), 0.95 (major isomer, s, 6.7H), 0.94 (minor isomer, s, 2.3H), 0.13 (major isomer, s, 2.3H), 0.13 (major isomer, s, 2.3H), 0.11 (minor isomer, s, 0.7H), 0.10 (minor isomer, s, 0.7H). $^{13}$C NMR (101 MHz, $CD_2Cl_2$) δ 139.3, 139.2, 137.0, 136.73, 136.71, 136.1, 133.7, 133.4, 133.3, 133.2, 128.6, 128.4, 128.3, 128.23, 128.19, 128.04, 128.01, 126.8, 126.64, 126.61, 126.58, 126.52, 126.46, 126.41, 126.38, 126.34, 126.26, 126.23, 126.18, 126.14, 126.11, 83.4, 82.2, 81.7, 80.1, 79.1, 78.6, 75.4, 75.24, 75.20, 74.8, 73.8, 73.6, 72.3, 72.28, 72.25, 71.9, 71.3, 71.2, 70.3, 69.8, 68.7, 43.04, 42.99, 42.0, 41.7, 33.9, 33.7, 26.09, 26.06, 26.0, 24.7, 23.1, 21.6, 21.4, 18.23, 18.20, -4.0, -4.1, -4.3, -4.4. IR (film) ṽ 3470, 3306, 2952, 2927, 2856, 1462, 1373, 1253, 1088, 973, 853, 837, 816, 777, 753, 640, 475 cm$^{-1}$. HRMS (ESI$^+$) Calcd for $C_{43}H_{54}NaO_5Si$ [M+Na]$^+$: 701.3632. Found: 701.3641.

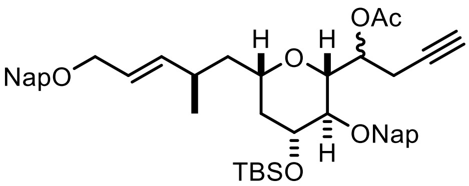


**Acetate S2.** Pyridine (650 µL, 8.04 mmol), $Ac_2O$ (180 µL, 1.90 mmol) and DMAP (24 mg, 197 µmol) were added at room temperature to a solution of alcohol **16** (654 mg, 0.963 mmol) in $CH_2Cl_2$ (6.5mL). After stirring for 5 h, the reaction was quenched with sat. aq. $NaHCO_3$ (10 mL), the layers were separated and the aqueous layer was extracted with $CH_2Cl_2$ (3 × 10 mL). The combined organic phases were dried over $Na_2SO_4$, the solvent was removed under reduced pressure, and the residue was purified by flash chromatography (fine silica, hexanes/*tert*-butyl methyl ether 3:1) to provide the title compound as an inseparable mixture of diastereomers in form of a colorless oil (dr ≈ 3:1, 625 mg, 90%). $^1$H NMR (400 MHz, $CD_2Cl_2$) δ 7.94 – 7.75 (m, 8H), 7.61 – 7.42 (m, 6H), 5.80 – 5.61 (m, 1H), 5.62 – 5.48 (m, 1H), 5.42 – 5.32 (m, 1H), 5.13 (major isomer, d, *J* = 11.5 Hz, 0.75H), 5.07 (minor isomer, d, *J* = 11.2 Hz, 0.25H), 4.86 (major isomer, d, *J* = 11.5 Hz, 0.75H), 4.69 – 4.61 (m, 2H), 4.57 (minor isomer, d, *J* = 10.9 Hz, 0.25H), 4.09 – 4.00 (m, 2H), 3.96 – 3.78 (m, 1H), 3.57 – 3.17 (m, 3H), 2.70 – 2.29 (m, 3H), 2.08 – 1.90 (m, 4H), 1.90 – 1.81 (m, 1H), 1.71 – 1.23 (m, 3H), 1.07 (minor isomer, d, *J* = 6.8 Hz, 0.7H), 1.04 (major isomer, d, *J* = 6.8 Hz, 2.3H), 0.95 (minor isomer, s, 2.3H), 0.95 (major isomer, s, 6.7H), 0.31 – -0.21 (m, 6H). $^{13}$C NMR (151 MHz, $CD_2Cl_2$) δ 170.5, 170.4, 139.4, 139.0, 136.74, 136.70, 136.6, 136.5, 133.73, 133.71, 133.70, 133.66, 133.4, 133.33, 133.32, 128.5, 128.4, 128.34, 128.34, 128.33, 128.32, 128.31, 128.29, 128.26, 128.20, 128.19, 128.18, 128.17, 128.03, 128.00, 127.99, 126.8, 126.72, 126.67, 126.58, 126.57, 126.47, 126.46, 126.44, 126.38, 126.34, 126.29, 126.24, 126.23, 126.18, 126.17, 126.16, 81.1, 80.5, 80.1, 79.5, 79.2, 77.2, 75.54, 75.48, 75.2, 74.9, 74.0, 73.6, 72.2, 71.9, 71.3, 71.2, 70.9, 70.1, 69.9, 43.1, 42.7, 41.8, 41.6, 33.9, 33.8, 26.09, 26.07, 21.6, 21.33, 21.25, 21.2, 20.6, 19.3, 18.22, 18.20, -4.0, -4.1, -4.3, -4.4. IR (film) ṽ 2952, 2927, 2856, 1741, 1371, 1236, 1091, 1031, 971, 852, 837, 816, 777, 753, 641, 476 cm$^{-1}$. HRMS (ESI$^{+/-}$) Calcd for $C_{45}H_{56}NaO_6Si$ $[M+Na]^+$: 743.3738. Found: 743.3739.

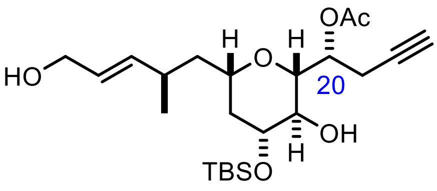


**Diol 17.** 2,3-Dichloro-5,6-dicyano-1,4-benzoquinone (652 mg, 2.87 mmol) was added to a solution of acetate **S2** (518 mg, 718 µmol) in $CH_2Cl_2$ (50 mL) and pH 7 phosphate buffer (0.05 M, 13 mL). The mixture was vigorously stirred for 2 h before it was poured into sat. aq. $NaHCO_3$ (50 mL). The aqueous layer was extracted with $CH_2Cl_2$ (5 × 20 mL), the combined organic phases were washed with brine and dried over $Na_2SO_4$, the solvent was removed under reduced pressure, and the residue was purified by flash chromatography (fine silica, hexanes/*tert*-butyl methyl ether 3:1 → 1:1) to provide the title compound as a colorless oil (214 mg, 67%).

Analytical and spectral data of the major diastereoisomer **17**: $[\alpha]_{20}^{D}$ = –31.5 (c = 1.00, $CH_2Cl_2$). $^1$H NMR (400 MHz, MeOD) δ 5.62 (dtd, *J* = 15.3, 5.8, 0.8 Hz, 1H), 5.46 (ddt, *J* = 15.3, 8.5, 1.4 Hz, 1H), 5.38 (ddd,

$J$ = 7.9, 5.6, 2.7 Hz, 1H), 4.01 (dd, $J$ = 5.7, 1.4 Hz, 2H), 3.57 (ddd, $J$ = 11.0, 8.1, 5.2 Hz, 1H), 3.38 (tdd, $J$ = 11.7, 3.1, 1.9 Hz, 1H), 3.26 (dd, $J$ = 9.9, 2.7 Hz, 1H), 3.17 (dd, $J$ = 9.9, 8.2 Hz, 1H), 2.67 – 2.54 (m, 2H), 2.50 – 2.40 (m, 1H), 2.25 (t, $J$ = 2.7 Hz, 1H), 2.08 (s, 3H), 1.80 (ddd, $J$ = 12.9, 5.3, 1.9 Hz, 1H), 1.50 (ddd, $J$ = 14.1, 9.8, 4.4 Hz, 1H), 1.36 – 1.22 (m, 2H), 1.01 (d, $J$ = 6.8 Hz, 3H), 0.90 (s, 9H), 0.10 (s, 3H), 0.09 (s, 3H). $^{13}$C NMR (101 MHz, MeOD) δ 172.1, 138.2, 129.9, 81.4, 80.6, 75.7, 74.4, 74.2, 73.2, 70.9, 63.7, 43.7, 42.3, 34.5, 26.4, 21.6, 21.1, 19.8, 18.9, -4.2, -4.5. IR (film) ṽ 3427, 3312, 2953, 2928, 2857, 1730, 1373, 1251, 1084, 1034, 972, 838, 778, 636 cm$^{-1}$. HRMS (ESI$^+$) Calcd for $C_{23}H_{40}NaO_6Si$ [M+Na]$^+$: 463.2486. Found: 463.2486.

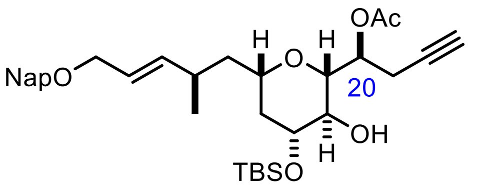


Analytical and spectral data of the minor diastereoisomer *epi*-**17** (66 mg, 21%): colorless oil; $[\alpha]_{20}^{D}$ = –42.0 (c = 1.00, $CH_2Cl_2$). $^1$H NMR (600 MHz, MeOD) δ 5.68 (dtd, $J$ = 15.4, 5.7, 0.9 Hz, 1H), 5.47 (ddt, $J$ = 15.3, 8.7, 1.5 Hz, 1H), 5.21 (ddd, $J$ = 9.3, 5.8, 1.9 Hz, 1H), 4.09 – 3.95 (m, 2H), 3.62 (ddd, $J$ = 11.1, 8.5, 5.2 Hz, 1H), 3.45 – 3.37 (m, 2H), 3.15 (dd, $J$ = 9.8, 8.5 Hz, 1H), 2.65 (ddd, $J$ = 16.0, 9.3, 2.7 Hz, 1H), 2.59 (ddd, $J$ = 16.0, 5.9, 2.8 Hz, 1H), 2.53 – 2.42 (m, 1H), 2.37 (t, $J$ = 2.7 Hz, 1H), 2.08 (s, 3H), 1.81 (ddd, $J$ = 12.8, 5.1, 1.9 Hz, 1H), 1.56 (ddd, $J$ = 14.0, 10.2, 3.8 Hz, 1H), 1.37 – 1.25 (m, 2H), 1.02 (d, $J$ = 6.9 Hz, 3H), 0.90 (s, 9H), 0.10 (s, 3H), 0.09 (s, 3H). $^{13}$C NMR (151 MHz, MeOD) δ 172.3, 137.7, 130.2, 80.5, 78.8, 75.6, 74.8, 72.5, 72.0, 71.3, 63.6, 43.9, 42.4, 34.7, 26.4, 21.8, 20.9, 20.8, 19.0, -4.2, -4.5. IR (film) ṽ 3425, 3311, 2952, 2927, 2856, 1725, 1375, 1248, 1083, 1034, 971, 836, 778, 671, 636 cm$^{-1}$. HRMS (ESI$^+$) Calcd for $C_{23}H_{40}NaO_6Si$ [M+Na]$^+$: 463.2486. Found: 463.2486.

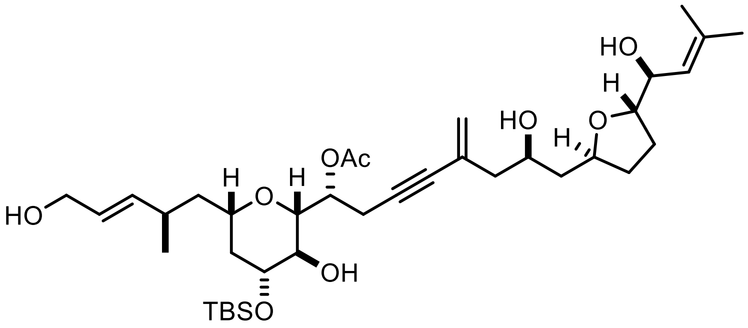


**Enyne 20.** A Schlenk flask was charged with diol **17** (141 mg, 320 µmol), alkenyl iodide **19** (100 mg, 273 µmol),[3] CuI (10.2 mg, 53.6 µmol), $Pd_2(dba)_3$ (12.3 mg, 13.4 µmol), and $PPh_3$ (14.0 mg, 53.4 µmol). Degassed THF (8.9 mL) and *N*-ethyldiisopropylamine (240 µL, 1.38 mmol) were added and the resulting mixture was stirred for 48 h. The reaction was quenched with sat. aq. $NH_4Cl$ (10 mL), the mixture was extracted with *tert*-butyl methyl ether (3 × 20 mL), and the combined organic layers were washed with brine and dried over $Na_2SO_4$. The solvent was removed under reduced pressure and the residue was purified by flash chromatography (silica, *tert*-butyl methyl ether + 1% $NEt_3$ → *tert*-butyl methyl ether/EtOAc 1:20 + 1% $NEt_3$) to provide the title compound as a pale yellow amorphous solid material (177 mg, 95%). $[\alpha]_{20}^{D}$ = –10.6 (c = 1.00, $CH_2Cl_2$). $^1$H NMR (400 MHz, $C_6D_6$) δ 5.77 (td, $J$ = 6.4, 2.9 Hz, 1H), 5.71 (dtd, $J$ = 15.3, 5.7,

0.8 Hz, 1H), 5.44 (d, $J$ = 2.2 Hz, 1H), 5.37 (ddt, $J$ = 15.3, 8.6, 1.5 Hz, 1H), 5.26 (dp, $J$ = 8.9, 1.5 Hz, 1H), 5.17 (dt, $J$ = 2.4, 1.3 Hz, 1H), 4.39 – 4.30 (m, 1H), 4.20 (dd, $J$ = 8.9, 6.8 Hz, 1H), 4.08 – 3.96 (m, 3H), 3.89 (q, $J$ = 7.0 Hz, 1H), 3.75 – 3.70 (m, 1H), 3.70 – 3.61 (m, 2H), 3.55 (dd, $J$ = 9.6, 3.0 Hz, 1H), 3.47 (d, $J$ = 3.8 Hz, 1H), 3.35 (ddt, $J$ = 11.9, 10.0, 2.6 Hz, 1H), 3.07 – 2.97 (m, 2H), 2.88 (dd, $J$ = 17.0, 6.1 Hz, 1H), 2.54 (ddd, $J$ = 14.7, 10.8, 4.1 Hz, 1H), 2.44 (dd, $J$ = 13.6, 7.5 Hz, 1H), 2.31 (dd, $J$ = 13.4, 5.2 Hz, 1H), 1.85 (s, 3H), 1.77 – 1.66 (m, 3H), 1.62 (d, $J$ = 1.5 Hz, 3H), 1.60 (d, $J$ = 1.4 Hz, 3H), 1.62 – 1.38 (m, 7H), 1.28 – 1.14 (m, 1H), 1.02 (s, 9H), 0.94 (d, $J$ = 6.8 Hz, 3H), 0.21 (s, 3H), 0.14 (s, 3H). $^{13}$C NMR (101 MHz, $C_6D_6$) δ 170.1, 136.5, 136.3, 129.7, 129.4, 125.0, 122.6, 87.7, 83.3, 83.0, 79.5, 79.2, 75.1, 73.9, 73.5, 72.6, 71.1, 69.9, 63.5, 45.9, 42.9, 42.0, 41.2, 33.5, 33.0, 27.6, 26.2, 26.0, 21.4, 20.9, 20.7, 18.7, 18.4, -4.1, -4.3. IR (film) ṽ 3388, 2928, 2856, 1738, 1439, 1371, 1249, 1086, 1030, 972, 837, 778, 542 cm$^{-1}$. HRMS (ESI$^+$) Calcd for $C_{37}H_{62}NaO_9Si$ [M+Na]$^+$: 701.4055. Found: 701.4053.

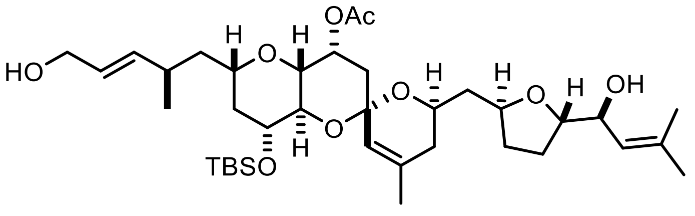


**Spiroketal 24.** PPTS (6.7 mg, 27 µmol) and (JohnPhos)Au(MeCN)$SbF_6$ (**21**, 20.7 mg, 26.8 µmol)[4] were added to a solution of enyne **20** (177 mg, 261 µmol) in $CH_2Cl_2$ (25 mL). After stirring for 15 min, the reaction was quenched with sat. aq. $NH_4Cl$ (5 mL) and sat. aq. $NaHCO_3$ (5 mL). The layers were separated and the aqueous phase was extracted with *tert*-butyl methyl ether (3 × 10 mL). The combined extracts were washed with brine and dried over $Na_2SO_4$. The solvent was removed under reduced pressure and the residue purified by flash chromatography (silica, hexanes/*tert*-butyl methyl ether 2:1 → 1:1) to provide the title compound as a colorless oil (137 mg, 77%). $[\alpha]_{20}^{D}$ = –44.3 (c = 1.00, $CH_2Cl_2$). $^1$H NMR (400 MHz, $C_6D_6$) δ 5.56 (dt, $J$ = 15.3, 5.5 Hz, 1H), 5.45 – 5.32 (m, 3H), 5.18 (d, $J$ = 1.5 Hz, 1H), 4.43 (dq, $J$ = 8.9, 6.1 Hz, 1H), 4.34 (dd, $J$ = 9.0, 7.4 Hz, 1H), 4.24 – 4.09 (m, 2H), 4.01 (q, $J$ = 7.2 Hz, 1H), 3.90 (dd, $J$ = 5.6, 1.5 Hz, 2H), 3.74 (ddd, $J$ = 10.9, 8.7, 5.3 Hz, 1H), 3.43 (ddt, $J$ = 11.8, 9.5, 2.7 Hz, 1H), 3.00 (dd, $J$ = 9.8, 3.2 Hz, 1H), 2.83 (s(br), 1H), 2.53 (tt, $J$ = 7.2, 3.3 Hz, 1H), 2.43 (dd, $J$ = 15.0, 3.0 Hz, 1H), 2.21 (dtd, $J$ = 11.7, 5.3, 2.9 Hz, 1H), 2.07 (s, 3H), 2.09 – 1.97 (m, 1H), 1.66 (d, $J$ = 1.5 Hz, 3H), 1.62 (d, $J$ = 1.6 Hz, 3H), 1.49 (s, 3H), 1.86 – 1.44 (m, 11H), 1.12 (ddd, $J$ = 14.7, 10.5, 3.9 Hz, 1H), 1.04 (s, 9H), 0.89 (d, $J$ = 6.7 Hz, 3H), 0.22 (s, 3H), 0.15 (s, 3H). $^{13}$C NMR (101 MHz, $C_6D_6$) δ 170.7, 136.8, 136.4, 136.1, 129.2, 125.2, 124.2, 95.1, 83.3, 77.3, 75.1, 74.1, 72.4, 71.6, 70.7, 68.4, 66.1, 63.4, 42.9, 41.9, 41.8, 38.5, 35.6, 33.34, 33.31, 28.1, 26.2, 26.0, 22.7, 21.41, 21.38, 18.8, 18.5, -3.9, -4.8. IR (film) ṽ 3419, 2928, 2857, 1737, 1441, 1379, 1249, 1108, 1075, 959, 836, 778 cm$^{-1}$. HRMS (ESI$^+$) Calcd for $C_{37}H_{62}NaO_9Si$ [M+Na]$^+$: 701.4055. Found: 701.4056.

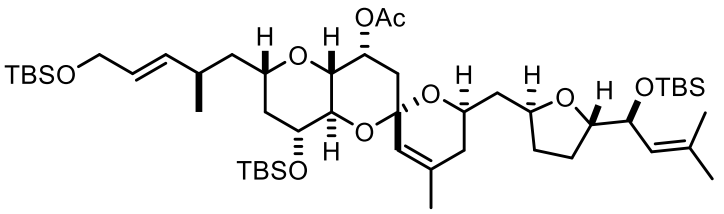


**Silyl ether 25.** 2,6-Lutidine (140 µL, 1.20 mmol) and TBSOTf (140 µL, 610 µmol) were added at –78 °C to a solution of spiroketal **24** (137 mg, 202 µmol) in $CH_2Cl_2$ (6.0 mL). After stirring for 1 h, the reaction was quenched upon addition of sat. aq. $NaHCO_3$ (10 mL). The layers were separated and the aqueous phase was extracted with $CH_2Cl_2$ (3 × 10 mL). The combined organic layers were washed with brine and dried over $Na_2SO_4$, the solvent was removed under reduced pressure, and the residue was purified by flash chromatography (fine silica, hexanes/*tert*-butyl methyl ether 20:1 + 1% $NEt_3$ $\rightarrow$ 15:1 + 1% $NEt_3$) to provide the title compound as a colorless oil (156 mg, 85%). $[\alpha]_{20}^{D}$ = –32.2 (c = 1.00, $CH_2Cl_2$). $^1$H NMR (400 MHz, $CD_2Cl_2$) δ 5.52 (dt, *J* = 15.3, 4.9 Hz, 1H), 5.44 (ddt, *J* = 15.3, 7.9, 1.1 Hz, 1H), 5.20 (p, *J* = 1.2 Hz, 1H), 5.14 (dp, *J* = 9.1, 1.4 Hz, 1H), 5.05 (q, *J* = 3.0 Hz, 1H), 4.34 – 4.19 (m, 2H), 4.11 (dd, *J* = 5.0, 1.5 Hz, 2H), 3.97 (ddt, *J* = 11.0, 9.1, 3.7 Hz, 1H), 3.87 (ddd, *J* = 7.9, 6.7, 5.6 Hz, 1H), 3.72 – 3.58 (m, 2H), 3.42 (dddd, *J* = 11.2, 9.0, 3.7, 1.9 Hz, 1H), 3.08 (dd, *J* = 9.4, 3.0 Hz, 1H), 2.38 (dtd, *J* = 9.6, 7.3, 4.8 Hz, 1H), 2.25 (dddd, *J* = 11.8, 7.9, 5.7, 3.3 Hz, 1H), 2.14 (dd, *J* = 15.0, 3.0 Hz, 1H), 2.05 (s, 3H), 1.97 – 1.87 (m, 2H), 1.87 – 1.79 (m, 2H), 1.76 (dd, *J* = 7.7, 3.2 Hz, 2H), 1.71 (d, *J* = 1.4 Hz, 3H), 1.68 (d, *J* = 0.9 Hz, 3H), 1.67 – 1.65 (m, 3H), 1.66 – 1.59 (m, 1H), 1.57 – 1.45 (m, 3H), 1.42 – 1.32 (m, 1H), 1.26 (ddd, *J* = 13.7, 9.7, 3.7 Hz, 1H), 0.97 (d, *J* = 6.7 Hz, 3H), 0.90 (s, 9H), 0.87 (s, 9H), 0.87 (s, 9H), 0.06 (s, 6H), 0.04 (s, 6H), 0.03 (s, 3H), 0.01 (s, 3H). $^{13}$C NMR (101 MHz, $CD_2Cl_2$) δ 171.2, 136.9, 136.4, 133.4, 129.0, 126.3, 123.6, 94.9, 83.0, 76.6, 75.3, 74.4, 73.0, 72.2, 70.5, 68.6, 65.2, 64.2, 43.0, 42.2, 41.7, 38.4, 35.6, 33.3, 32.8, 28.1, 26.2, 26.1, 22.8, 21.8, 21.4, 18.8, 18.7, 18.6, 18.5, -4.19, -4.22, -4.4, -4.89, -4.94. IR (film) ṽ 2953, 2928, 2856, 1739, 1462, 1379, 1248, 1108, 1069, 959, 834, 775, 666 cm$^{-1}$. HRMS (ESI$^+$) Calcd for $C_{49}H_{90}NaO_9Si_3$ $[M+Na]^+$: 929.5785. Found: 929.5785. The spectral data matched the literature.[3]

# COPIES OF SPECTRA

## **Compound 5** (literature known)[1]

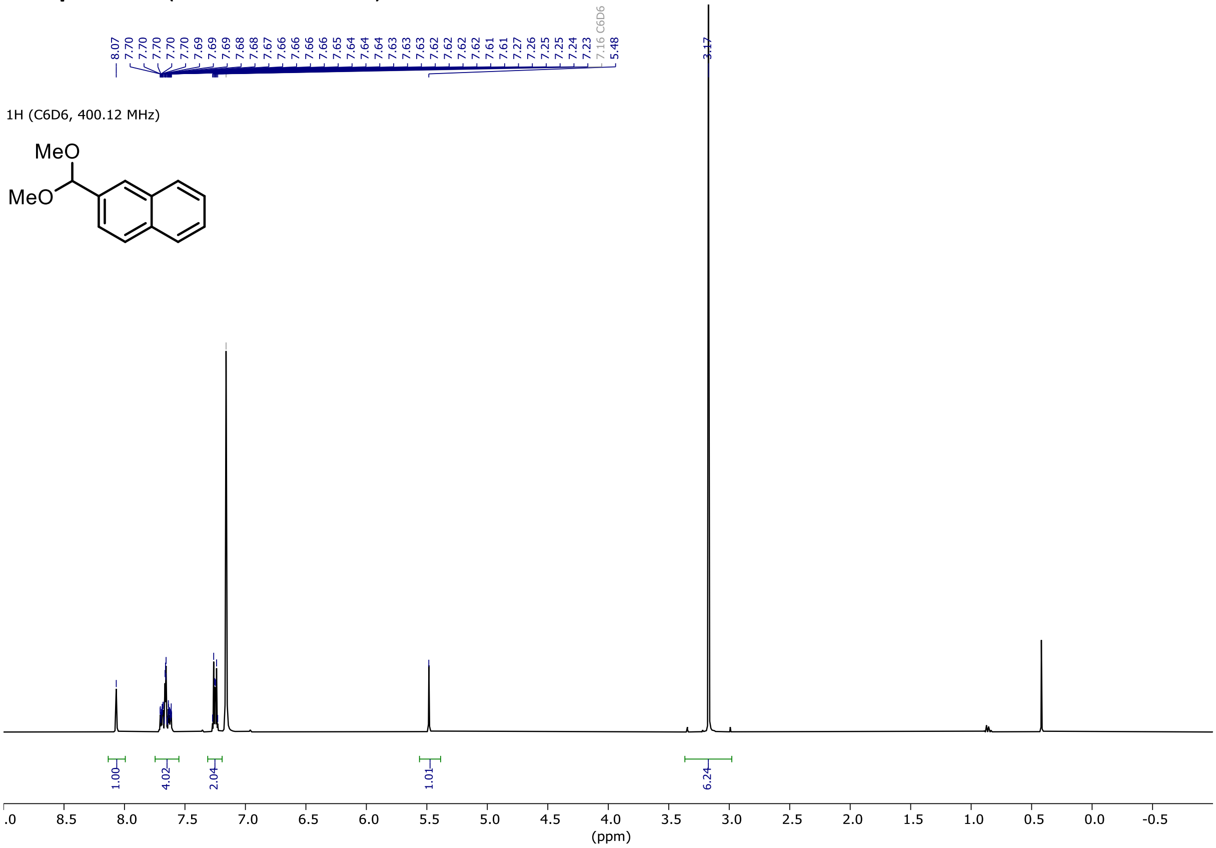


## **Compound 10** (literature known)[2]

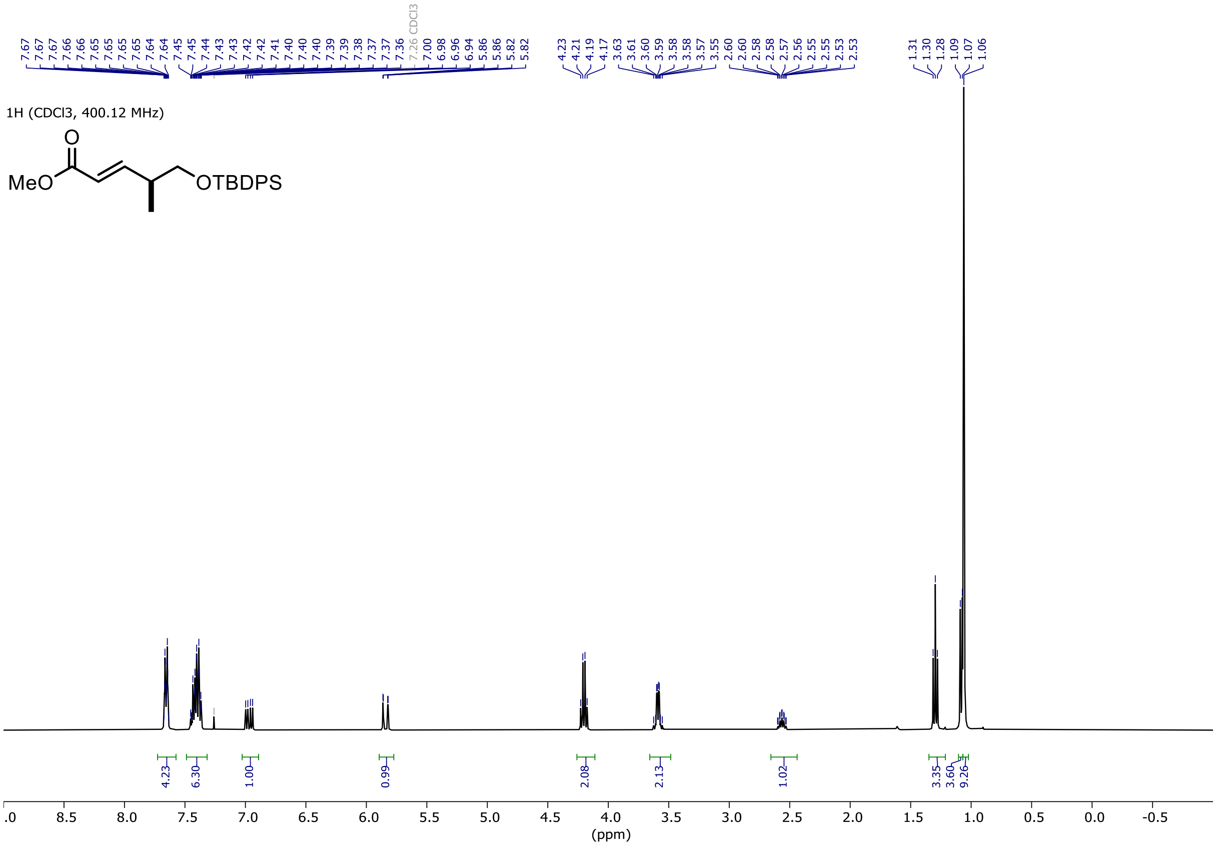

## Compound 6 (mixture of anomers)

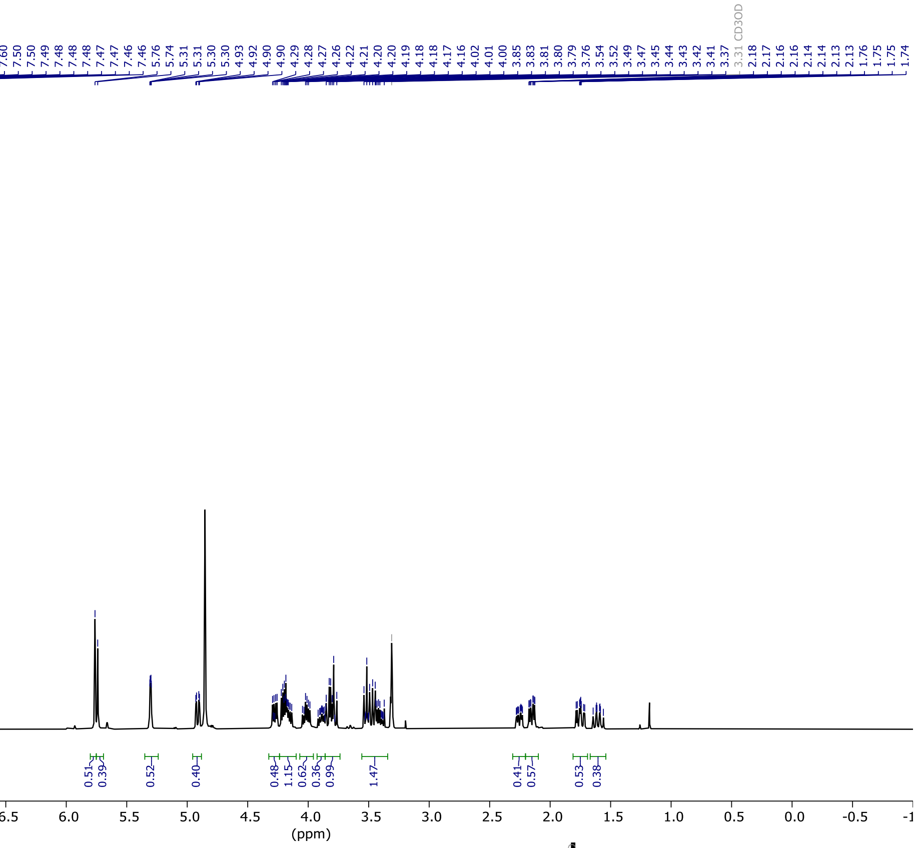


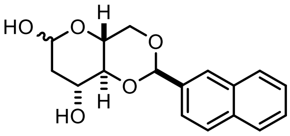


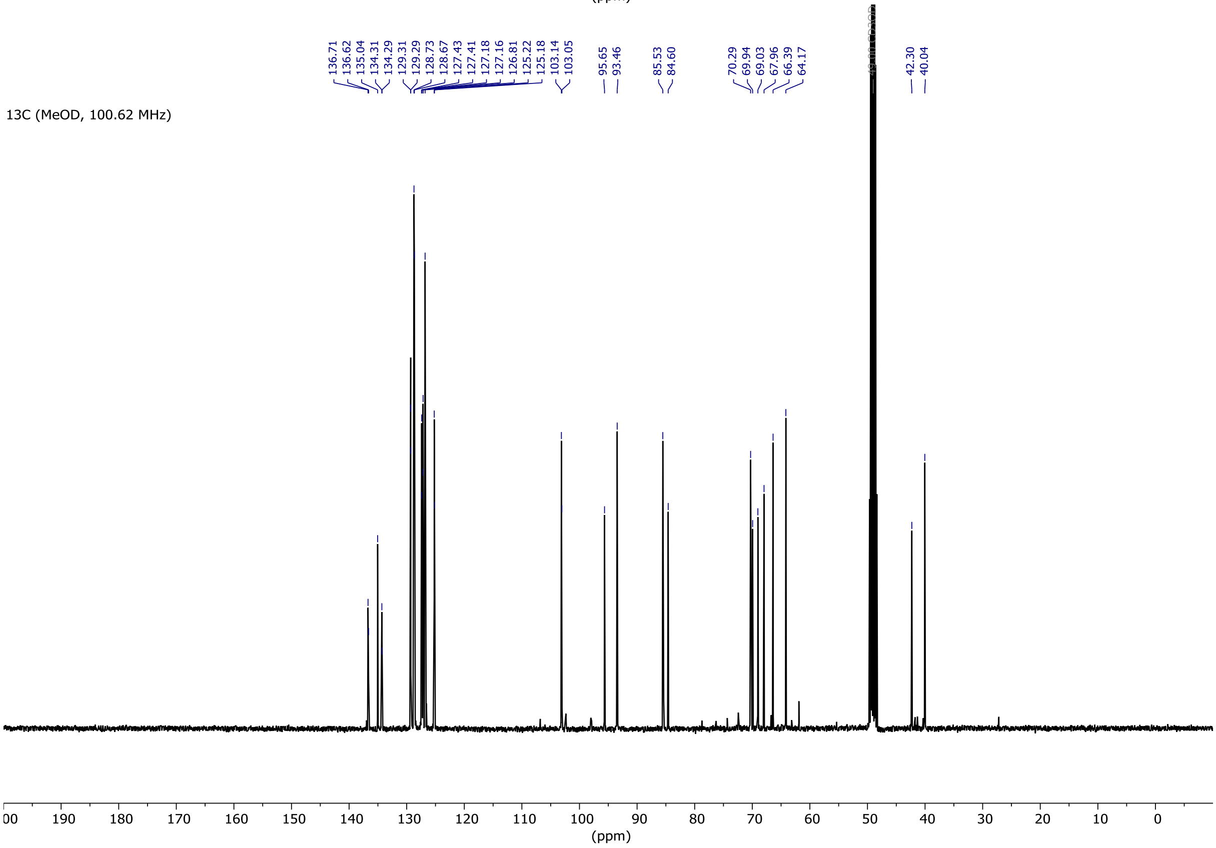

## Compound 8

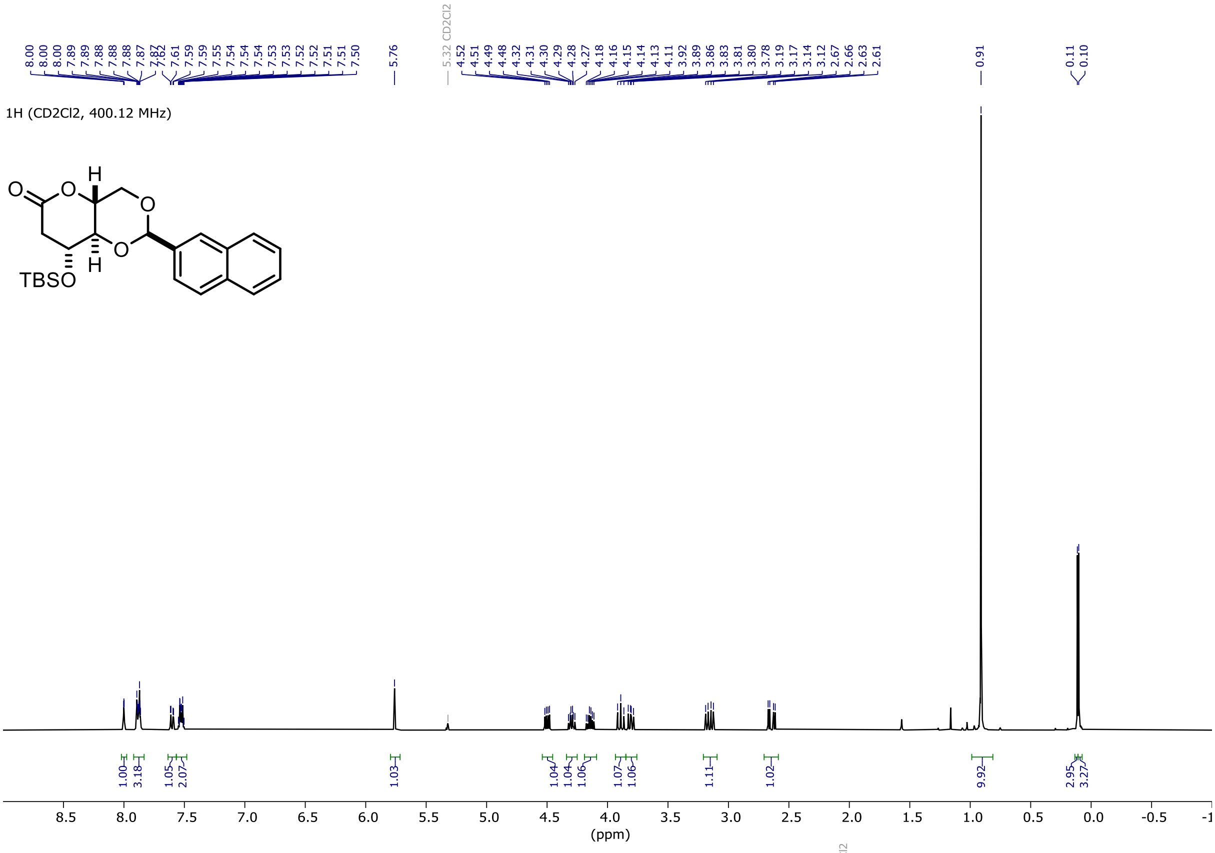
8.00 8.00 8.00 7.89 7.89 7.88 7.88 7.88 7.87 7.87 7.62 7.61 7.59 7.59 7.55 7.54 7.54 7.54 7.53 7.53 7.52 7.52 7.51 7.51 7.50
5.76
5.32 CD2Cl2
4.52 4.51 4.49 4.48 4.32 4.31 4.30 4.29 4.28 4.27 4.18 4.16 4.15 4.14 4.13 4.11 3.92 3.89 3.86 3.83 3.81 3.80 3.78 3.19 3.17 3.14 3.12 2.67 2.66 2.63 2.61
0.91
0.11 0.10
1H (CD2Cl2, 400.12 MHz)
1.00 3.18 1.05 2.07 1.03 1.04 1.04 1.06 1.07 1.06 1.11 1.02 9.92 2.95 3.27
8.5 8.0 7.5 7.0 6.5 6.0 5.5 5.0 4.5 4.0 3.5 3.0 2.5 2.0 1.5 1.0 0.5 0.0 -0.5
(ppm)
TBSO

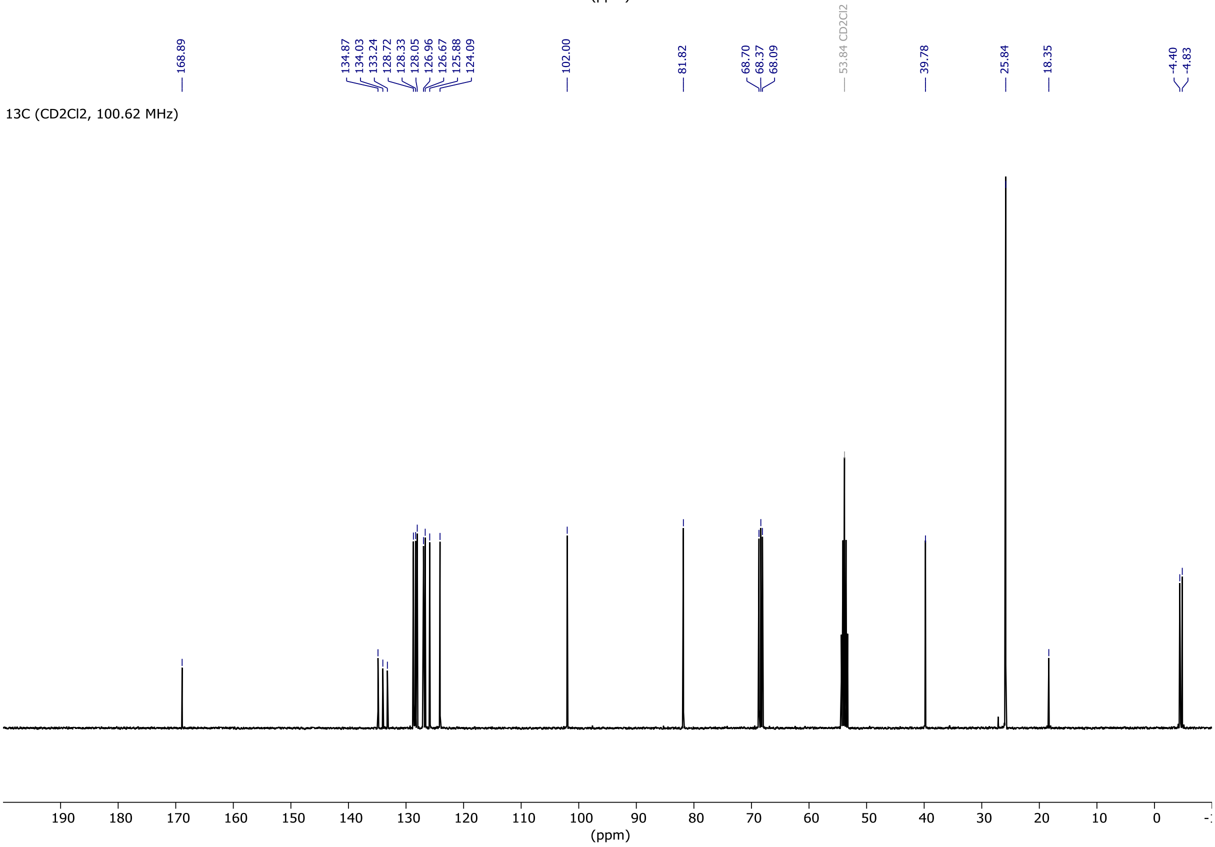
168.89
134.87 134.03 133.24 128.72 128.33 128.05 126.96 126.67 125.88 124.09
102.00
81.82
68.70 68.37 68.09
53.84 CD2Cl2
39.78
25.84
18.35
-4.40 -4.83
13C (CD2Cl2, 100.62 MHz)
190 180 170 160 150 140 130 120 110 100 90 80 70 60 50 40 30 20 10 0
(ppm)

## Compound 11

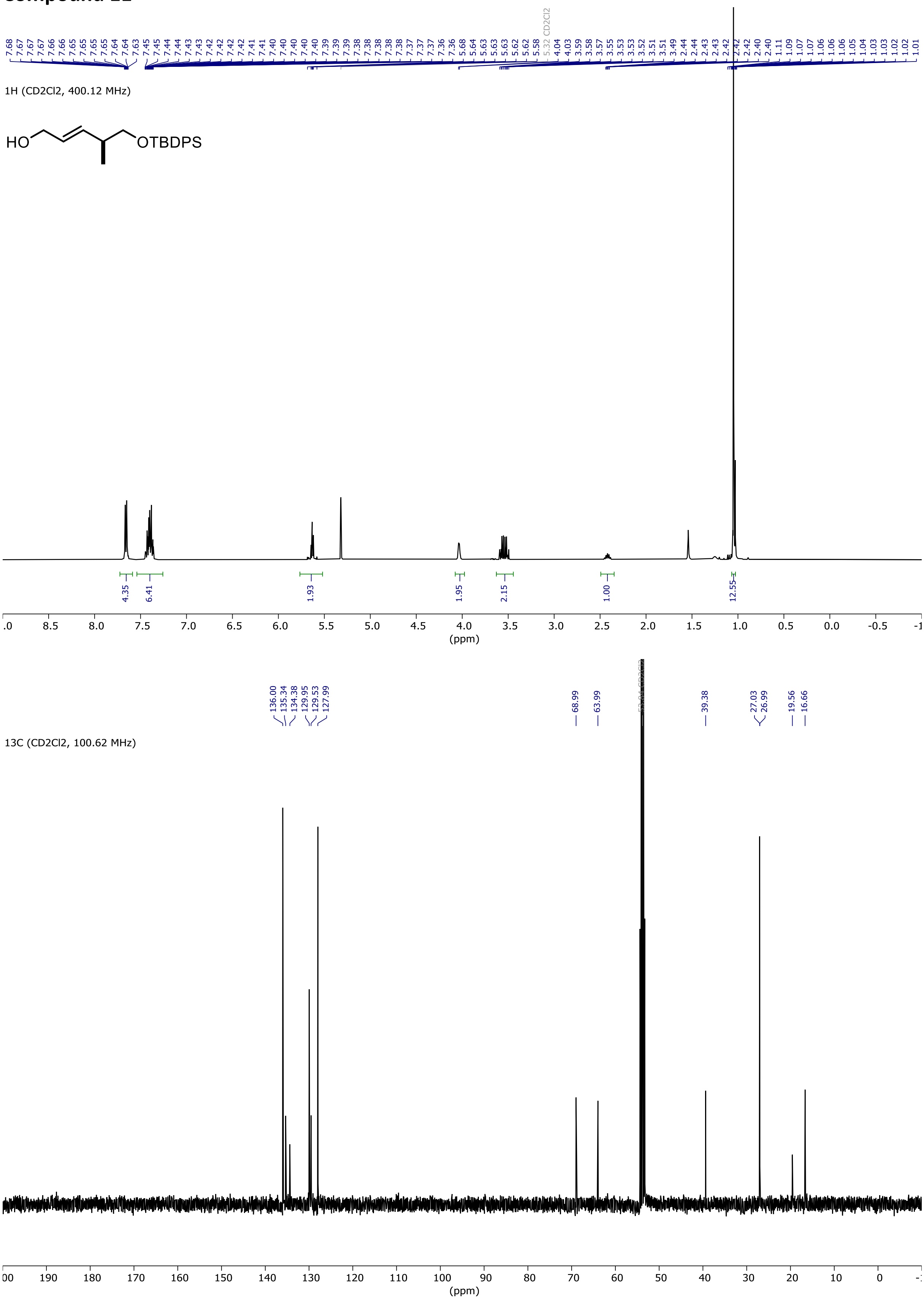
1H (CD2Cl2, 400.12 MHz)
HO
OTBDPS
4.35
6.41
1.93
1.95
2.15
1.00
12.55
(ppm)
13C (CD2Cl2, 100.62 MHz)
136.00
135.34
134.38
129.95
129.53
127.99
68.99
63.99
39.38
27.03
26.99
19.56
16.66
(ppm)

## Compound S1

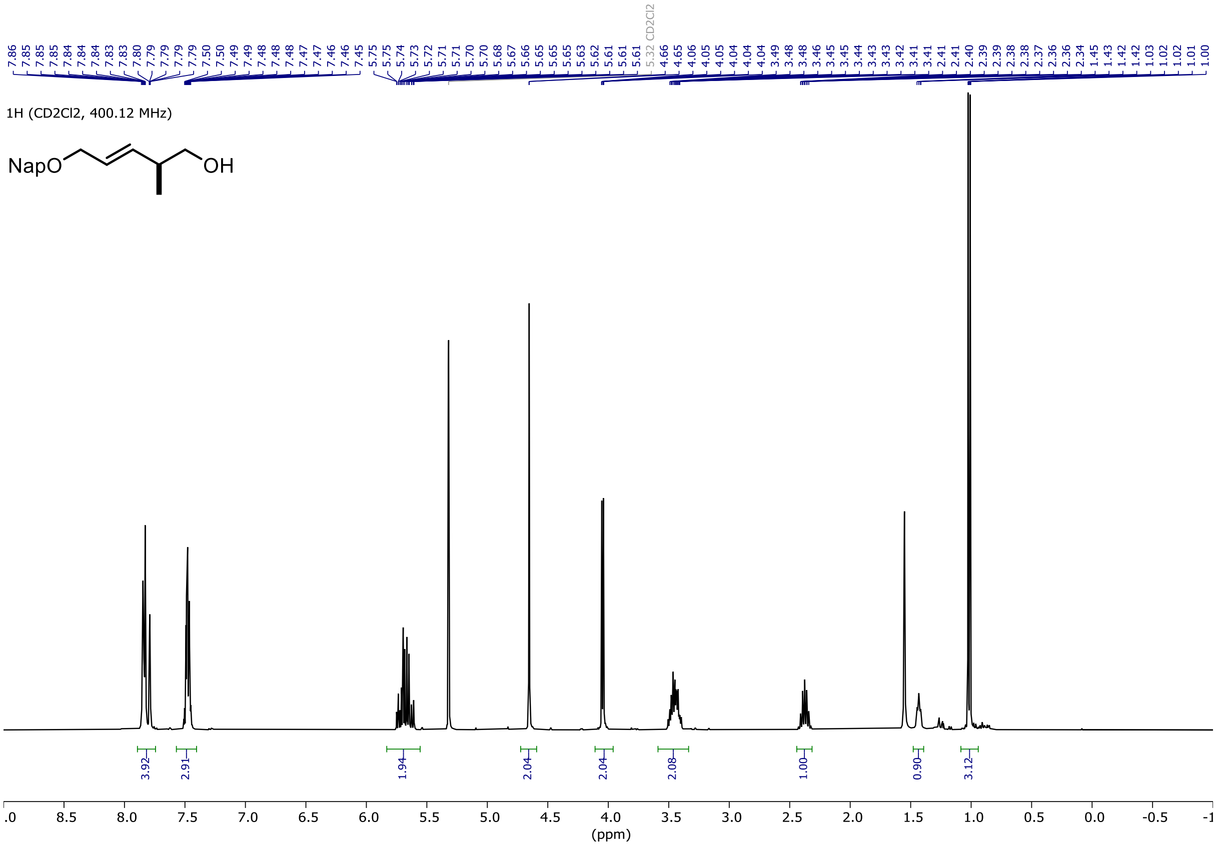
7.86 7.85 7.85 7.84 7.84 7.84 7.83 7.80 7.79 7.79 7.79 7.50 7.49 7.49 7.48 7.48 7.48 7.47 7.46 7.45 5.75 5.75 5.74 5.73 5.72 5.71 5.71 5.70 5.68 5.67 5.66 5.65 5.65 5.65 5.63 5.62 5.61 5.61 5.61 5.32 CD2Cl2 4.66 4.65 4.06 4.05 4.05 4.04 4.04 4.04 3.49 3.48 3.48 3.46 3.45 3.45 3.44 3.43 3.43 3.42 3.41 3.41 2.41 2.41 2.40 2.39 2.39 2.38 2.38 2.37 2.36 2.36 2.34 1.45 1.43 1.42 1.42 1.03 1.02 1.02 1.01 1.00
1H (CD2Cl2, 400.12 MHz)
NapO
OH
3.92 2.91 1.94 2.04 2.04 2.08 1.00 0.90 3.12
(ppm)

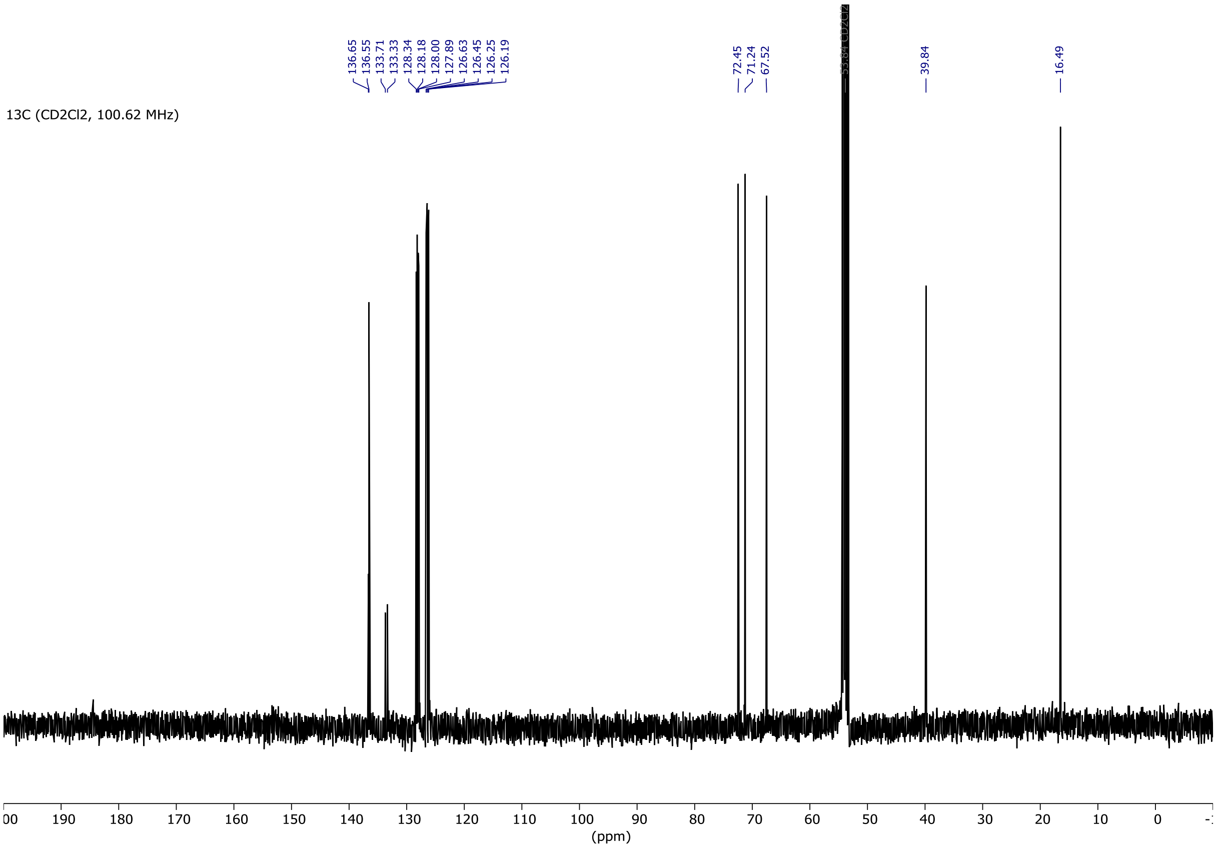
136.65 136.55 133.71 133.33 128.34 128.18 128.00 127.89 126.63 126.45 126.25 126.19
72.45 71.24 67.52
53.84 CD2Cl2
39.84
16.49
13C (CD2Cl2, 100.62 MHz)
(ppm)

## Compound 12

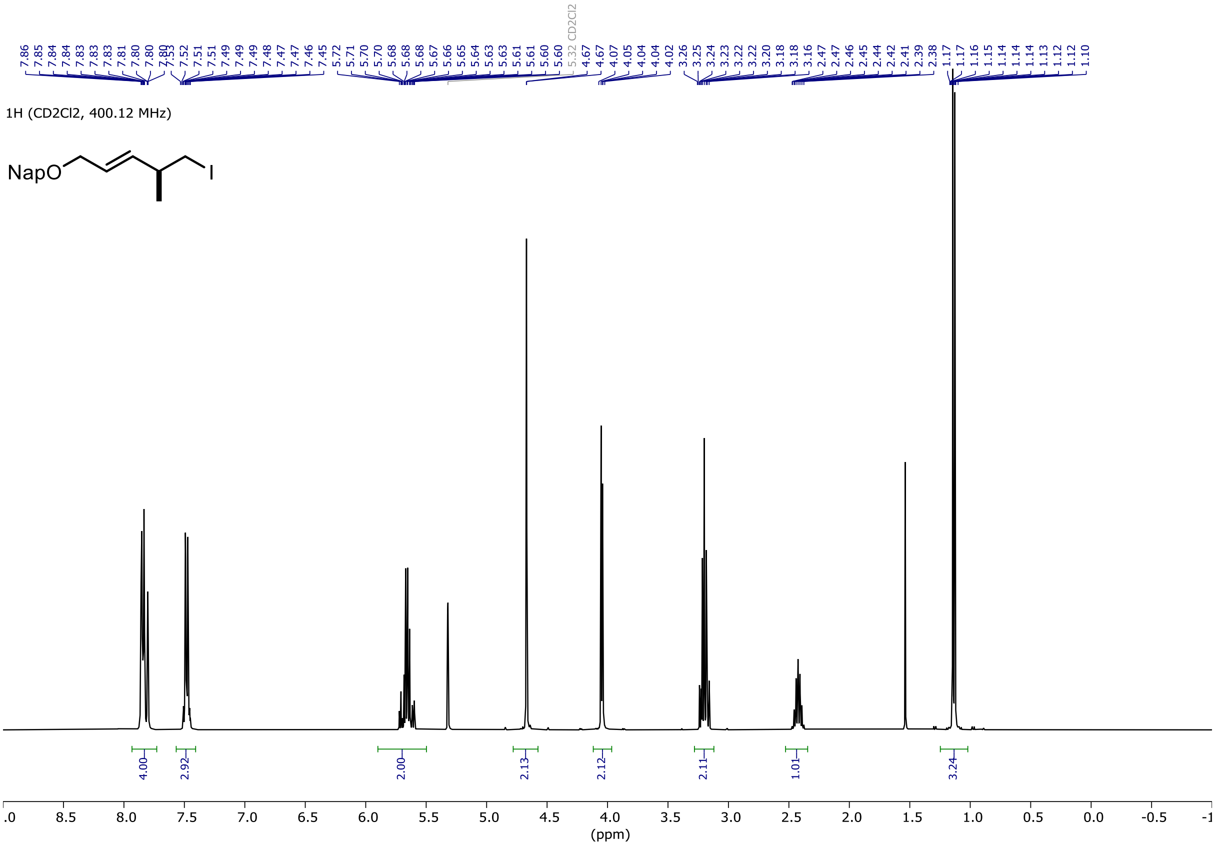

1H (CD2Cl2, 400.12 MHz)
NapO
I
7.86 7.85 7.84 7.84 7.83 7.83 7.83 7.81 7.80 7.80 7.80 7.53 7.52 7.51 7.51 7.49 7.49 7.49 7.48 7.47 7.47 7.46 7.45 5.72 5.71 5.70 5.70 5.68 5.68 5.68 5.67 5.66 5.65 5.65 5.64 5.63 5.63 5.61 5.61 5.60 5.60
5.32 CD2Cl2
4.67 4.67 4.07 4.05 4.04 4.04 4.02 3.26 3.25 3.24 3.23 3.22 3.22 3.20 3.18 3.18 3.16 2.47 2.47 2.46 2.45 2.44 2.42 2.41 2.39 2.38 1.17 1.17 1.16 1.15 1.14 1.14 1.14 1.13 1.12 1.12 1.10
4.00 2.92 2.00 2.13 2.12 2.11 1.01 3.24
(ppm)


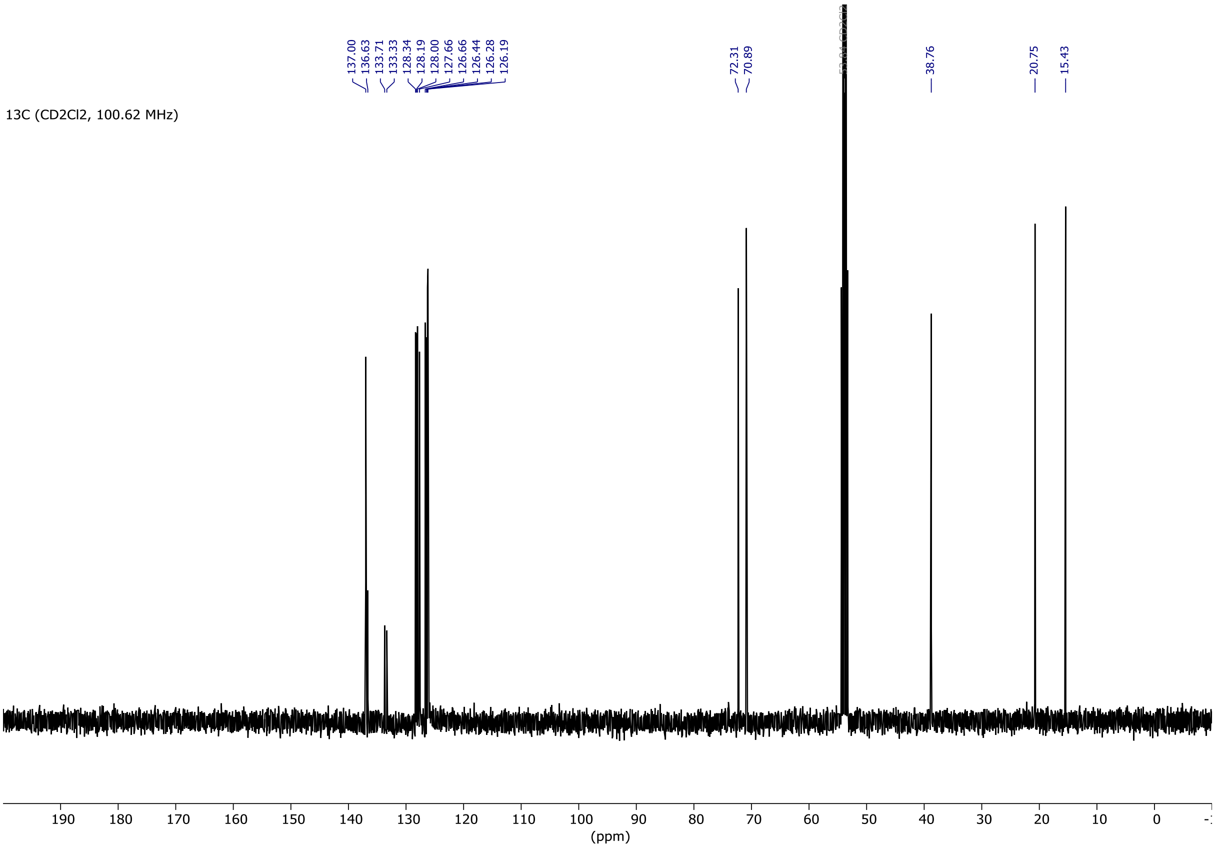

13C (CD2Cl2, 100.62 MHz)
137.00 136.63 133.71 133.33 128.34 128.19 128.00 127.66 126.66 126.44 126.28 126.19
72.31 70.89
38.76
20.75
15.43
(ppm)

## Compound 13 (mixture of anomers)

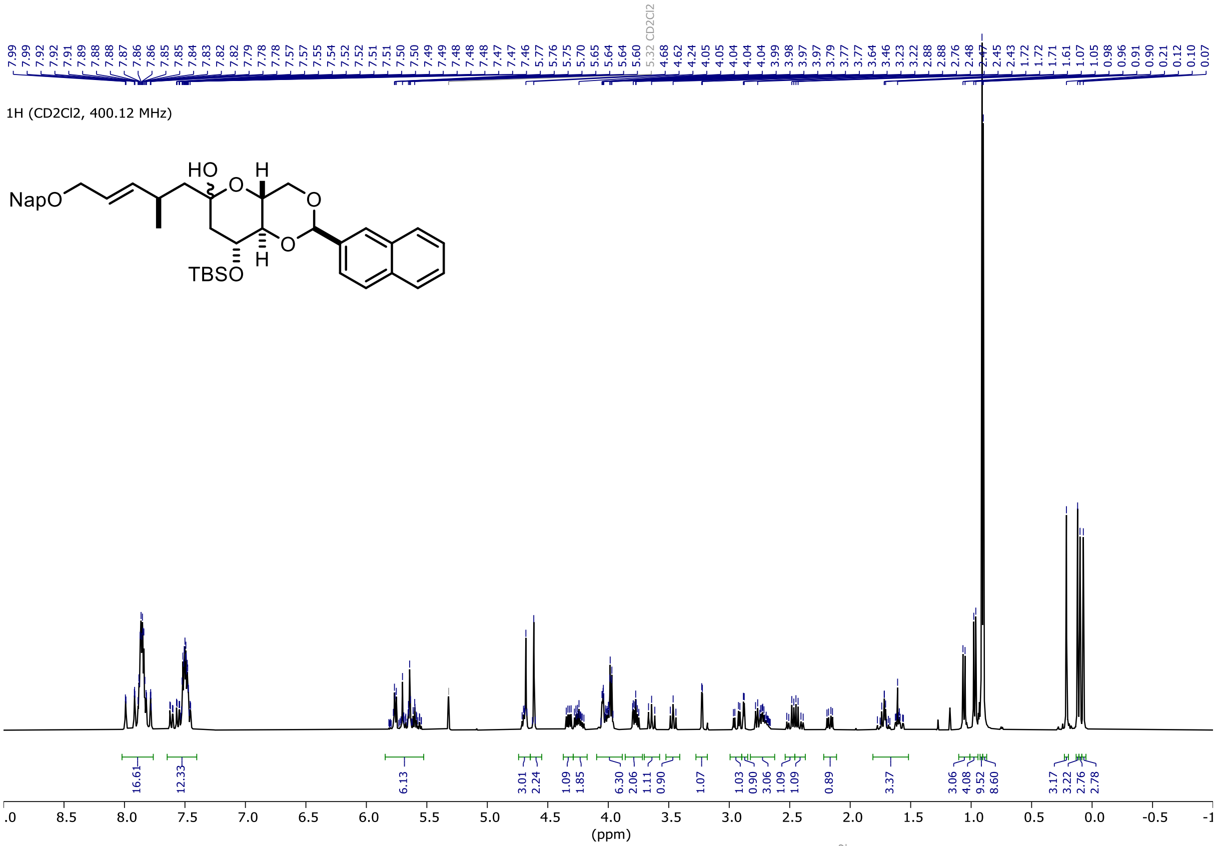


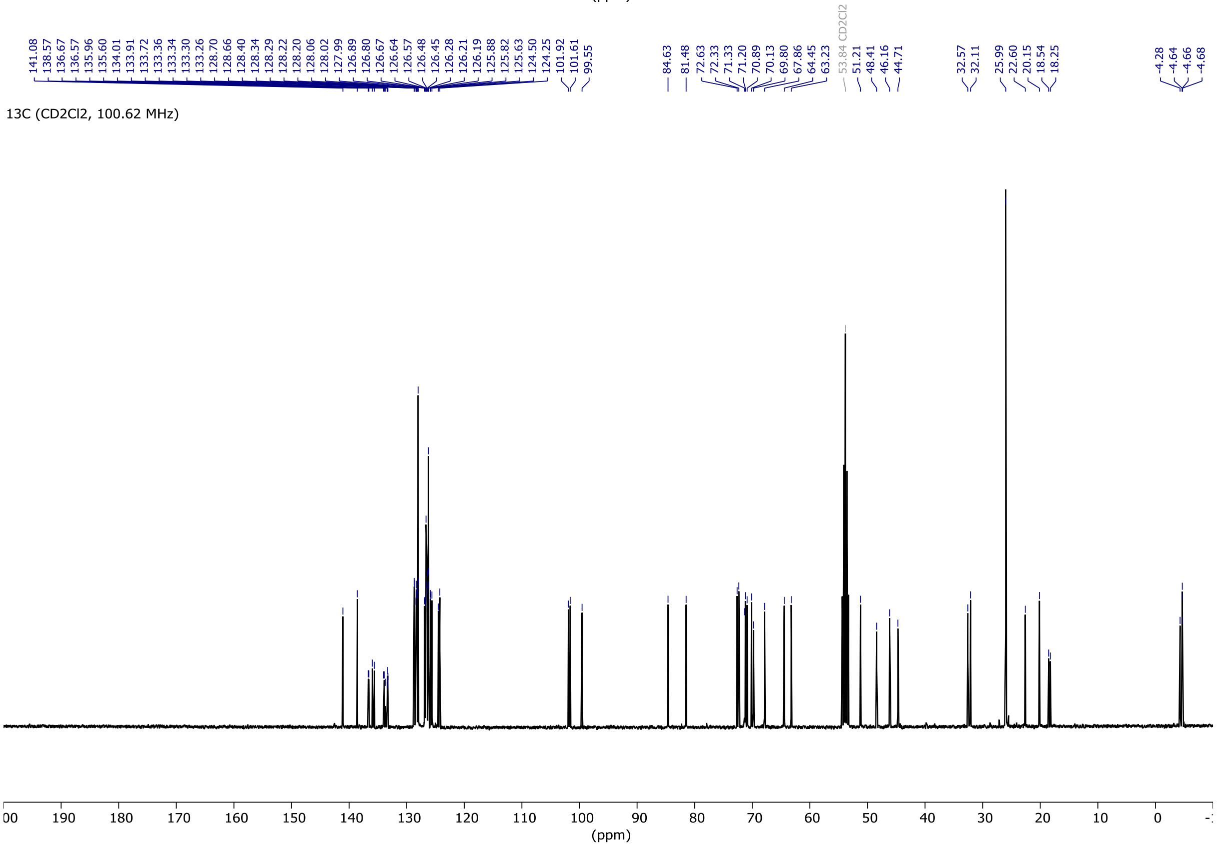

## Compound 14

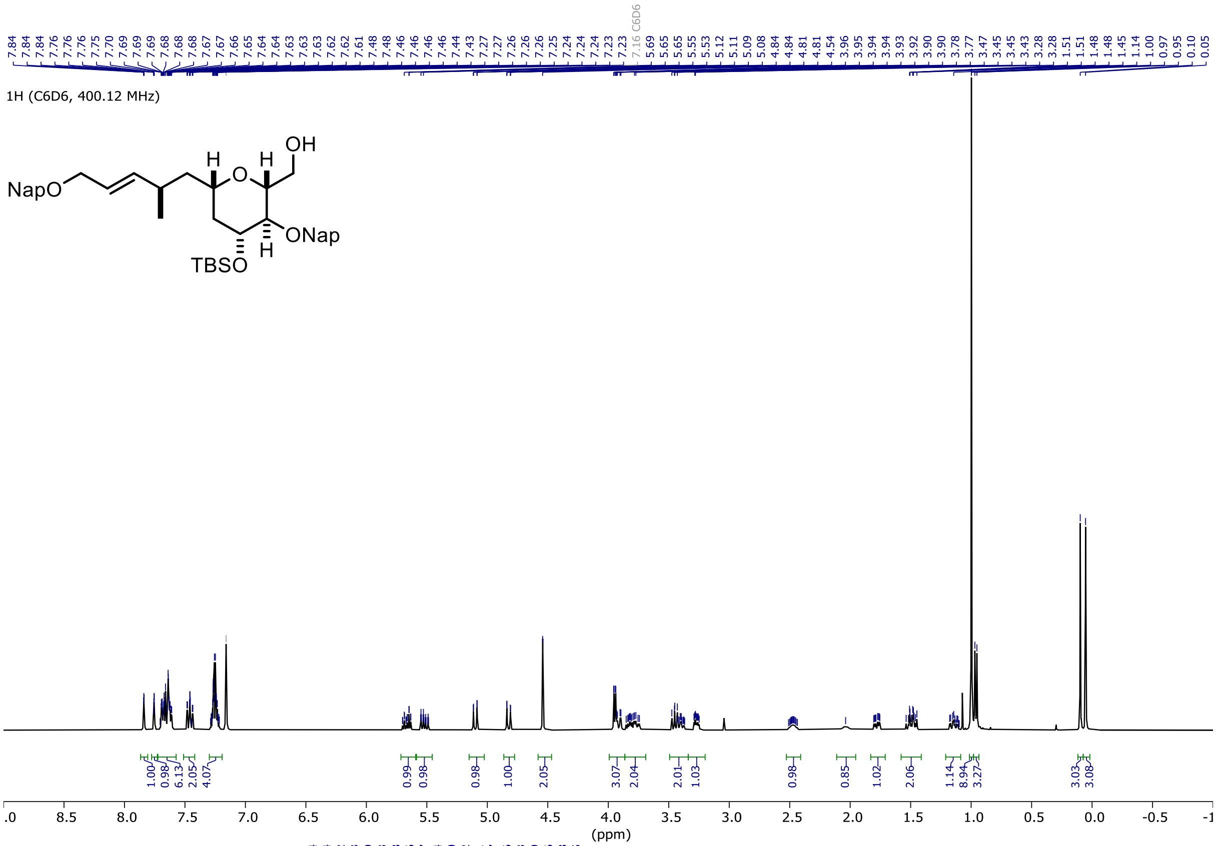

7.84 7.84 7.84 7.76 7.76 7.75 7.70 7.69 7.69 7.69 7.68 7.68 7.68 7.67 7.66 7.65 7.64 7.63 7.63 7.63 7.62 7.61 7.48 7.46 7.46 7.46 7.44 7.43 7.27 7.27 7.26 7.26 7.26 7.25 7.24 7.24 7.24 7.23 7.23 7.16 C6D6 5.69 5.65 5.65 5.55 5.53 5.12 5.11 5.09 5.08 4.84 4.84 4.81 4.54 3.96 3.95 3.94 3.94 3.93 3.92 3.90 3.90 3.78 3.77 3.47 3.45 3.45 3.43 3.28 3.28 1.51 1.51 1.48 1.48 1.45 1.14 1.00 0.97 0.95 0.10 0.05
1H (C6D6, 400.12 MHz)
1.00 0.98 6.13 2.05 4.07 0.99 0.98 0.98 1.00 2.05 3.07 2.04 2.01 1.03 0.98 0.85 1.02 2.06 1.14 8.94 3.27 3.03 3.08
(ppm)
OH
H
H
NapO
ONap
H
TBSO


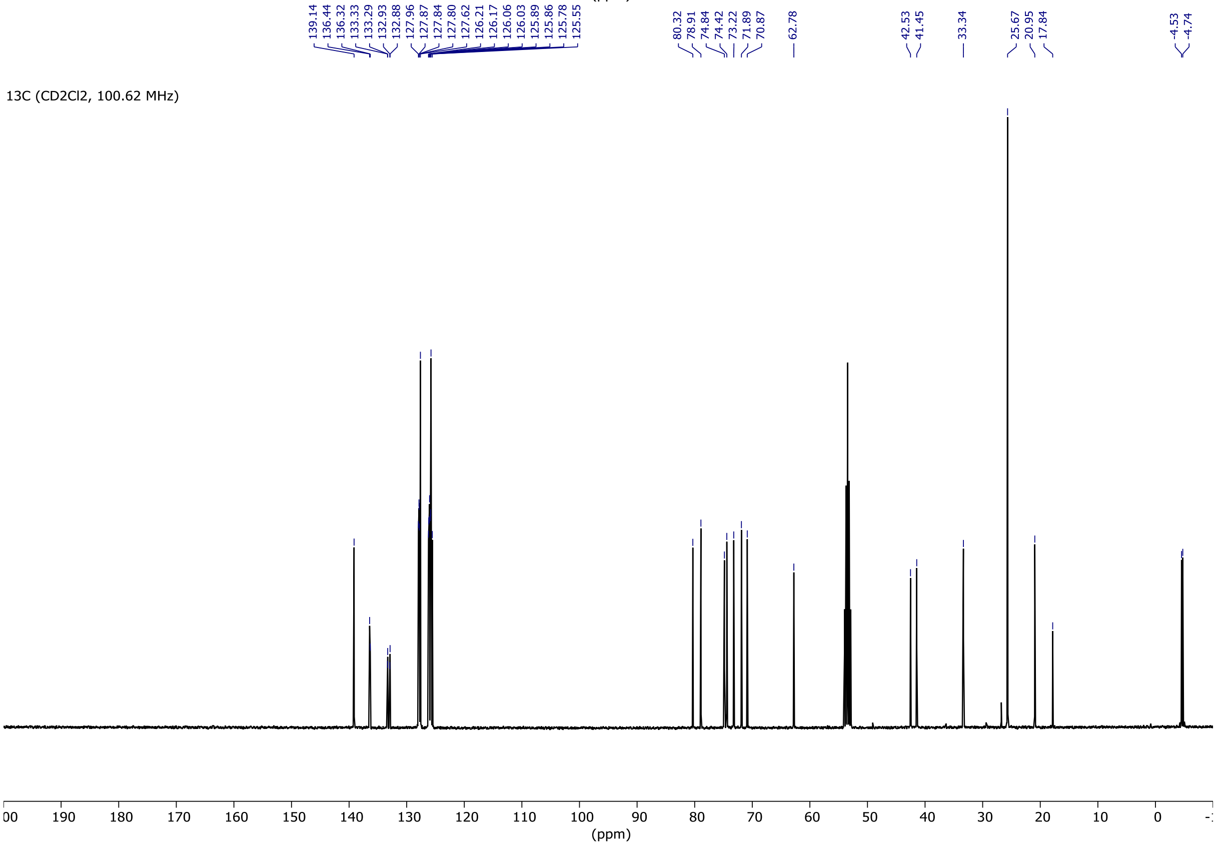

139.14 136.44 136.32 133.33 133.29 132.93 132.88 127.96 127.87 127.84 127.80 127.62 126.21 126.17 126.06 126.03 125.89 125.86 125.78 125.55
80.32 78.91 74.84 74.42 73.22 71.89 70.87 62.78 42.53 41.45 33.34 25.67 20.95 17.84 -4.53 -4.74
13C (CD2Cl2, 100.62 MHz)
(ppm)

## Compound 16 (mixture of diastereomers)

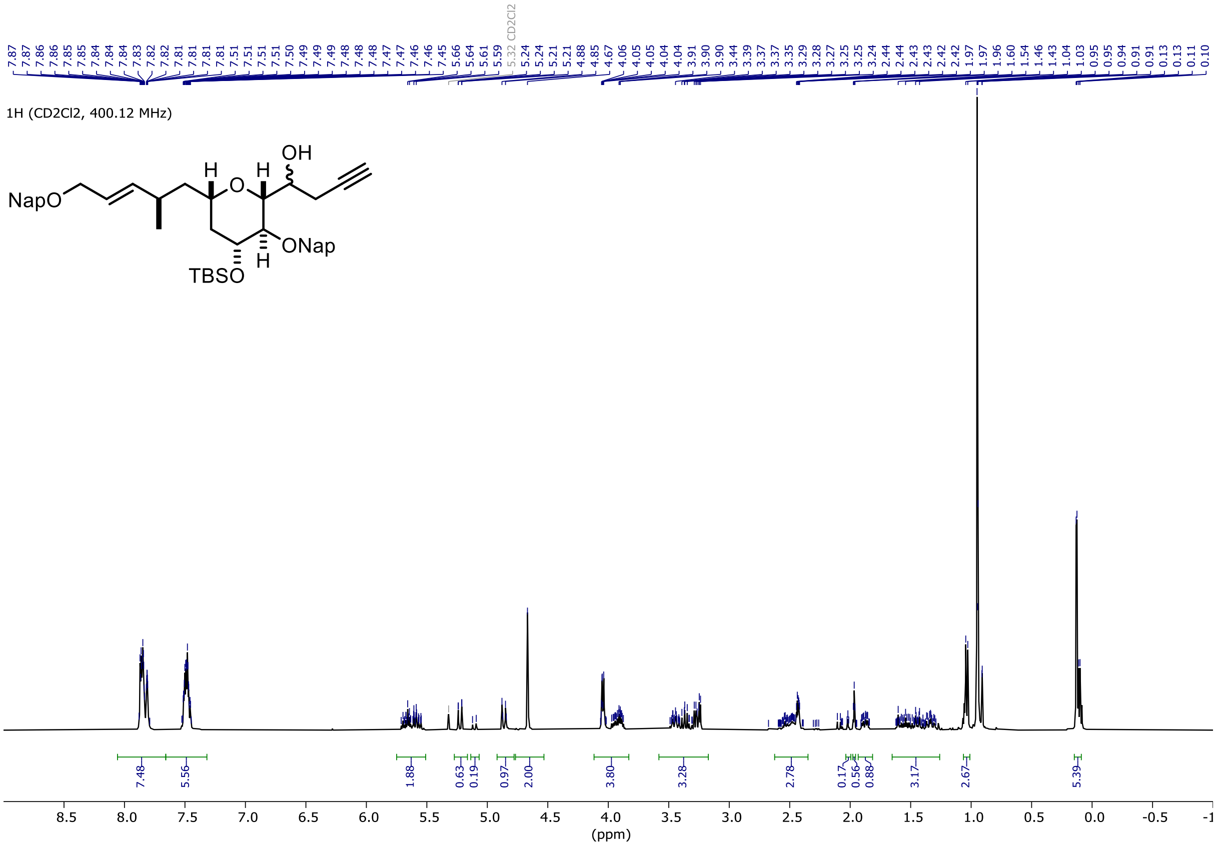


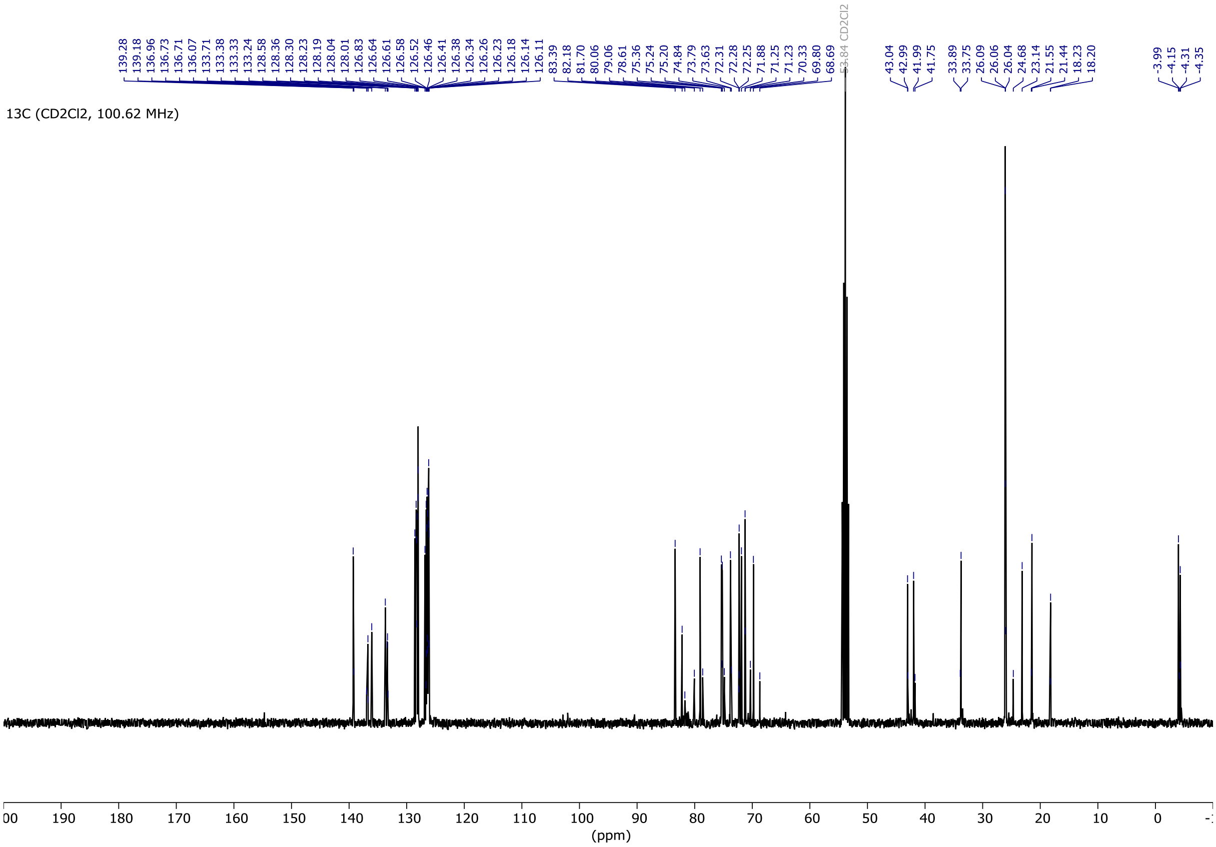

## Compound S2 (mixture of diastereomers)

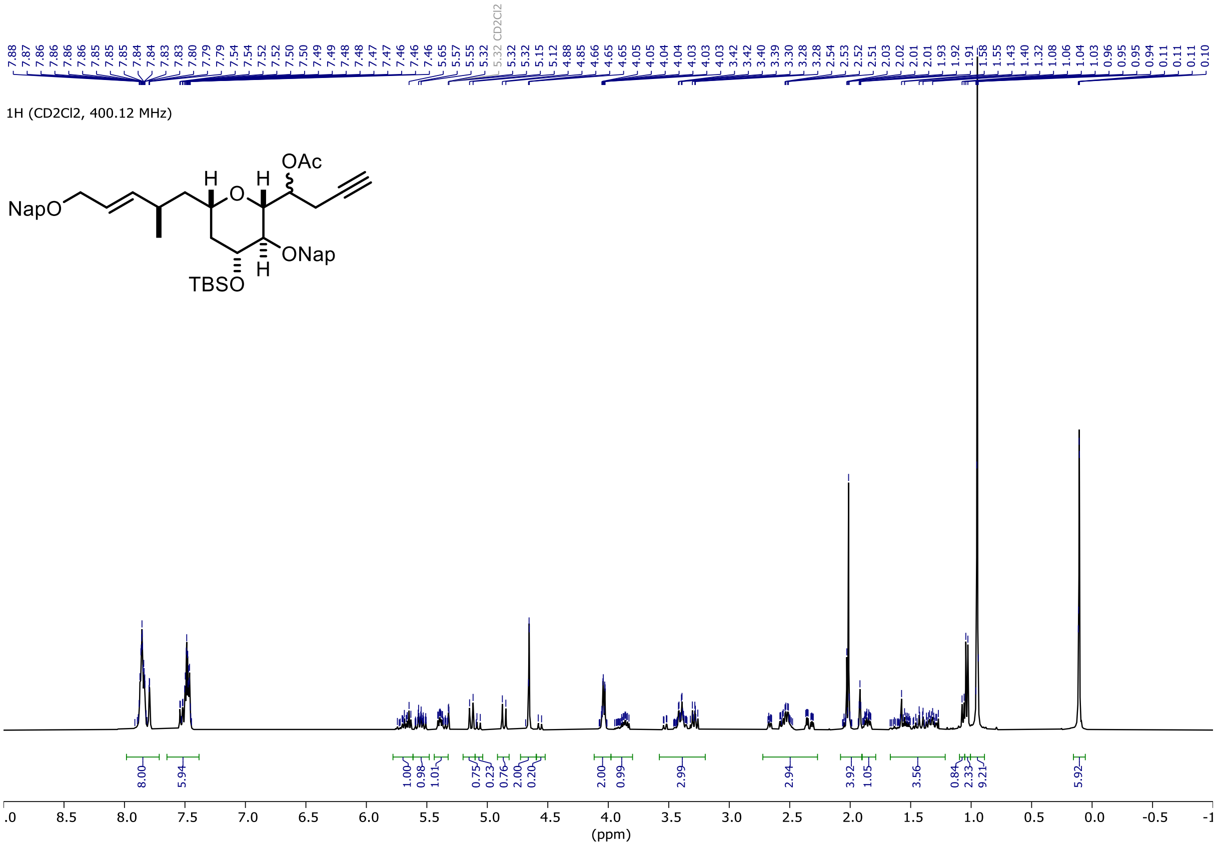


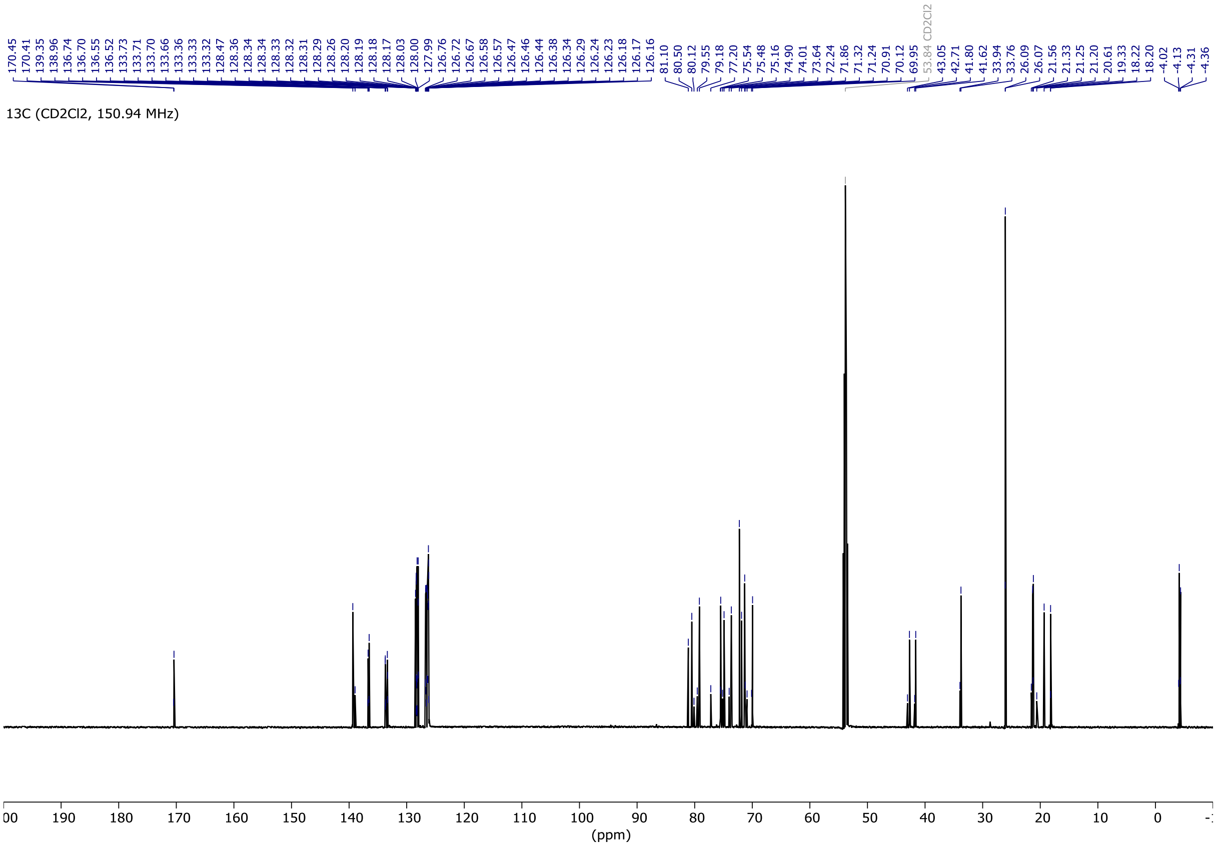

## Compound 17 (major diastereomer)

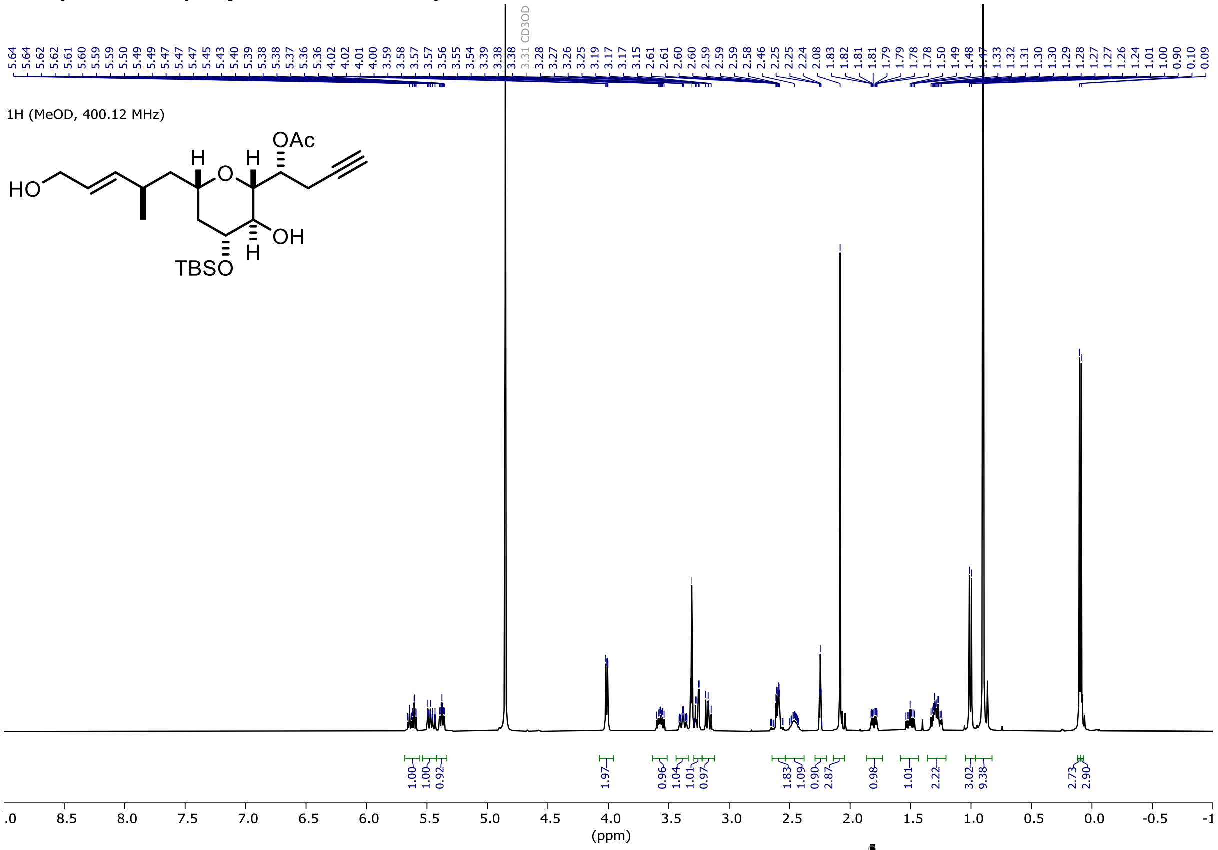


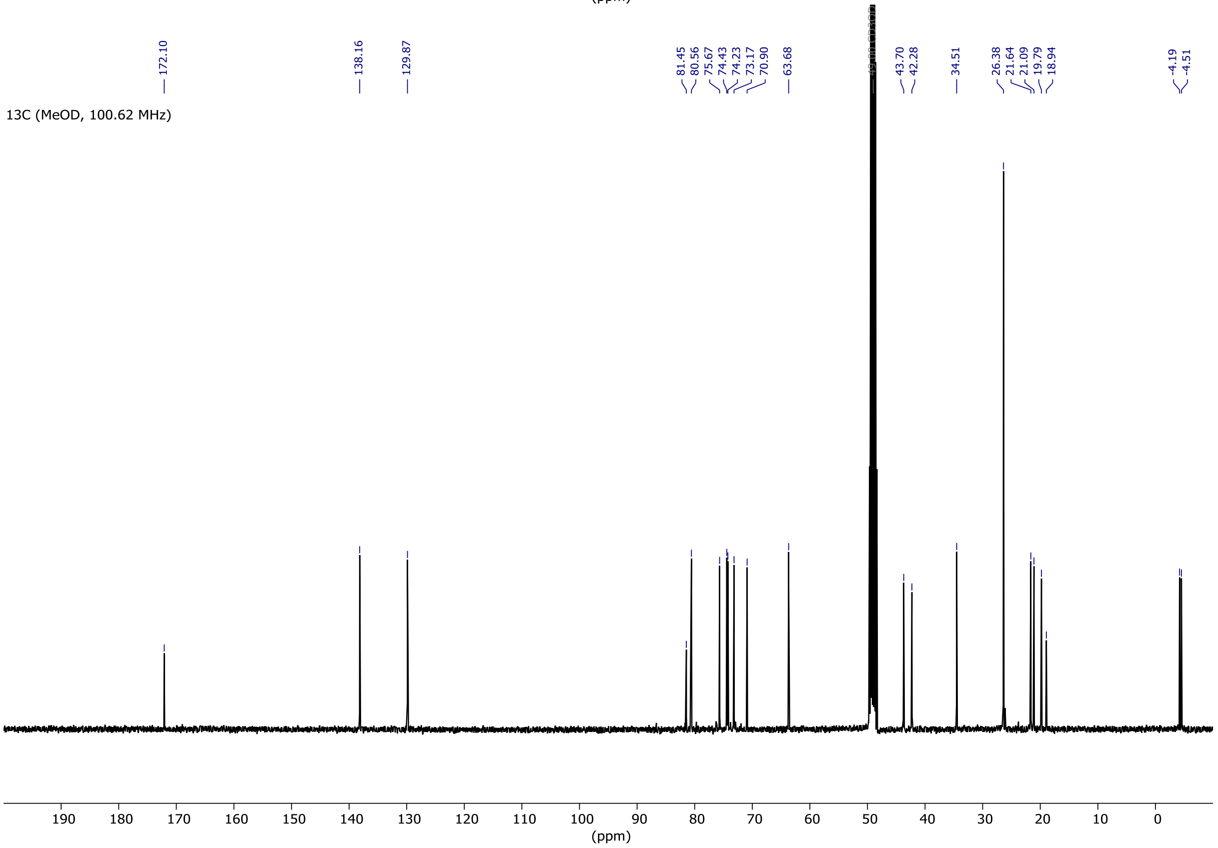

## Compound *epi*-17 (minor diastereomer)

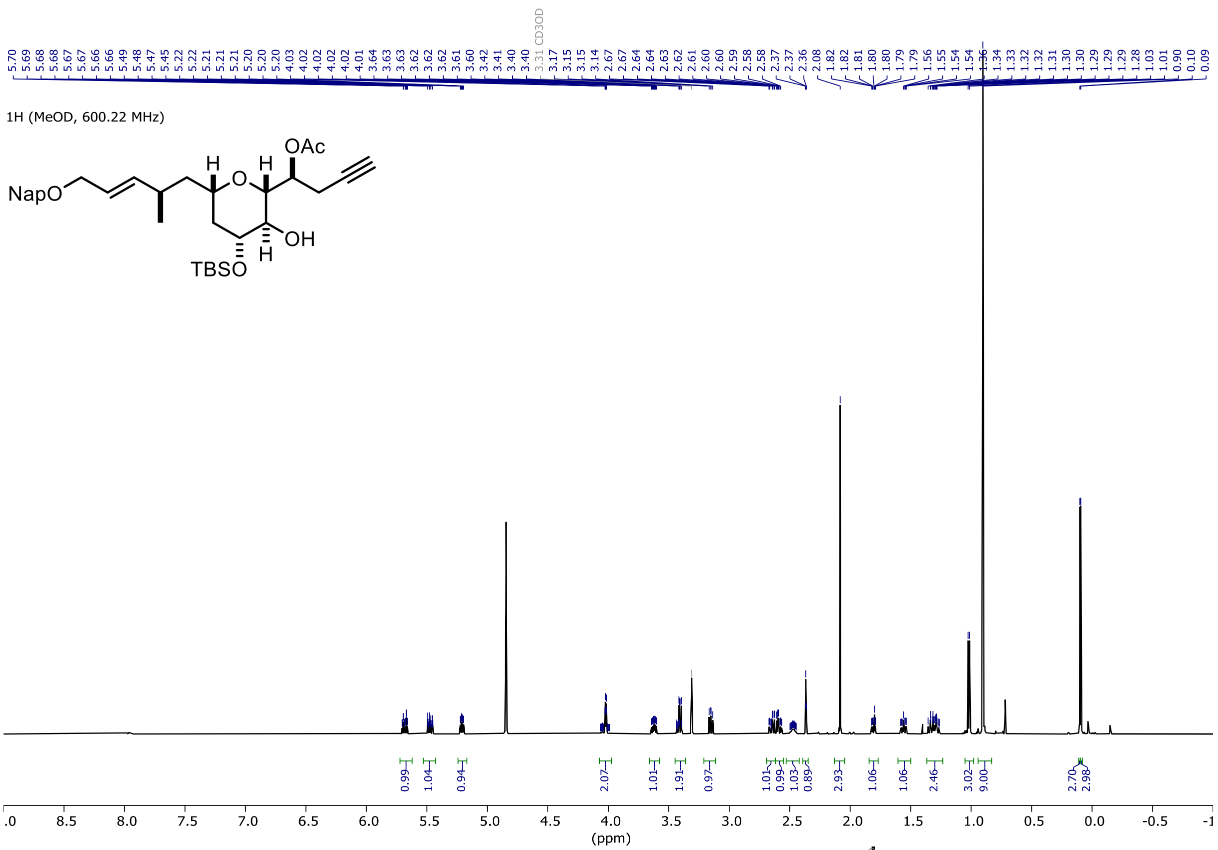


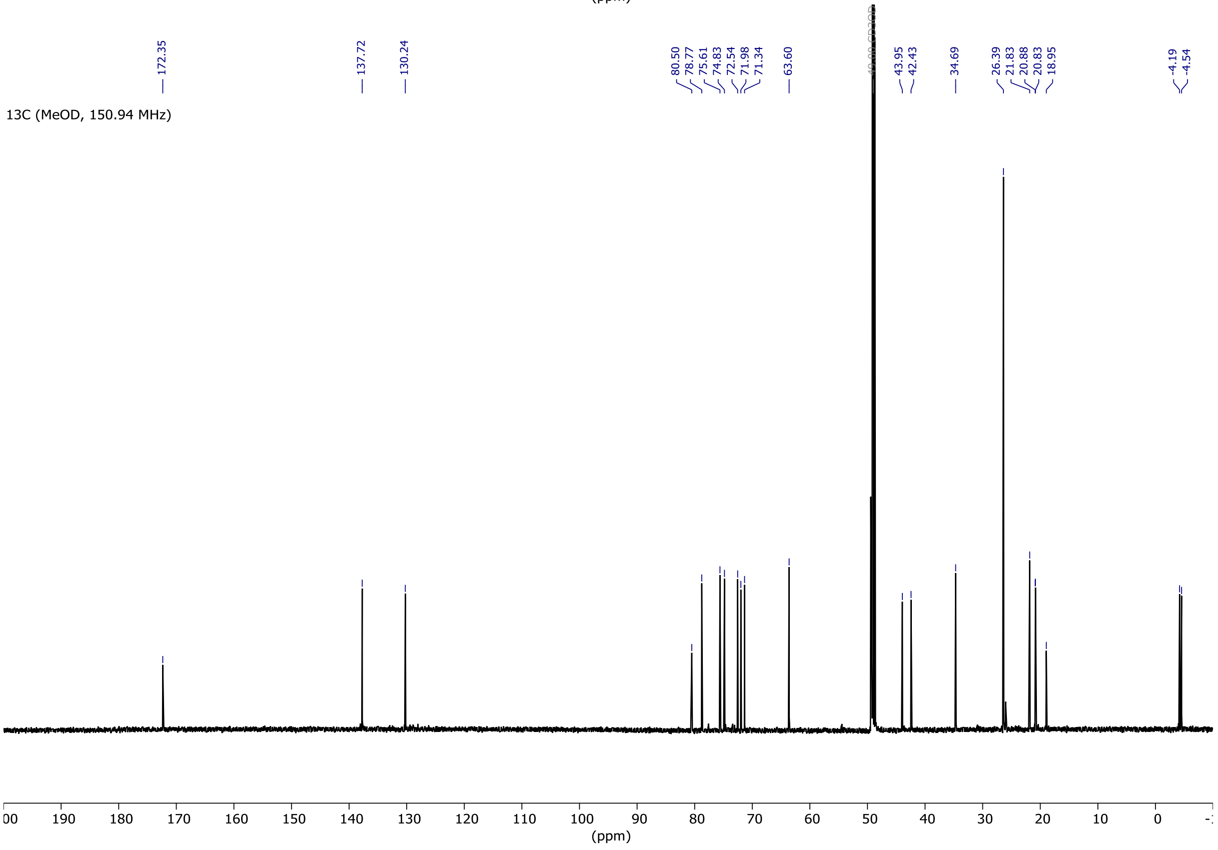

# Compound 20

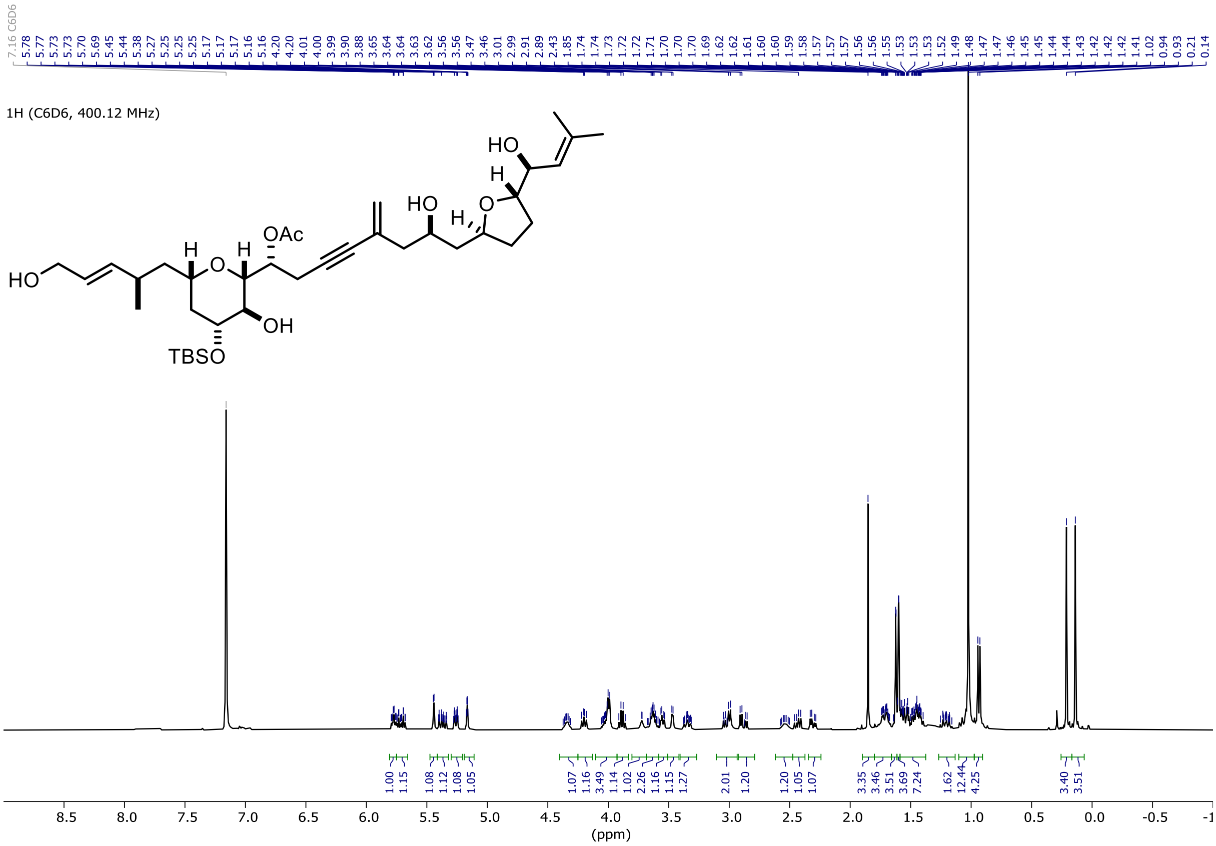
7.16 C6D6
5.78 5.77 5.73 5.73 5.70 5.69 5.45 5.44 5.38 5.27 5.25 5.25 5.25 5.17 5.17 5.17 5.16 4.20 4.20 4.01 4.00 3.99 3.90 3.88 3.65 3.64 3.64 3.63 3.62 3.56 3.56 3.47 3.46 3.01 2.99 2.91 2.89 2.43 1.85 1.74 1.74 1.73 1.72 1.72 1.71 1.70 1.70 1.70 1.69 1.62 1.62 1.61 1.60 1.60 1.59 1.58 1.57 1.57 1.57 1.56 1.56 1.55 1.53 1.53 1.53 1.53 1.52 1.49 1.48 1.47 1.47 1.46 1.45 1.45 1.44 1.44 1.43 1.42 1.42 1.42 1.41 1.02 0.94 0.93 0.21 0.14
1H (C6D6, 400.12 MHz)
HO
H
HO
H
O
OAc
H
O
H
HO
OH
TBSO
1.00 1.15 1.08 1.12 1.08 1.05 1.07 1.16 3.49 1.14 1.02 2.26 1.16 1.15 1.27 2.01 1.20 1.20 1.05 1.07 3.35 3.46 3.51 3.69 7.24 1.62 12.44 4.25 3.40 3.51
8.5 8.0 7.5 7.0 6.5 6.0 5.5 5.0 4.5 4.0 3.5 3.0 2.5 2.0 1.5 1.0 0.5 0.0 -0.5 -1
(ppm)

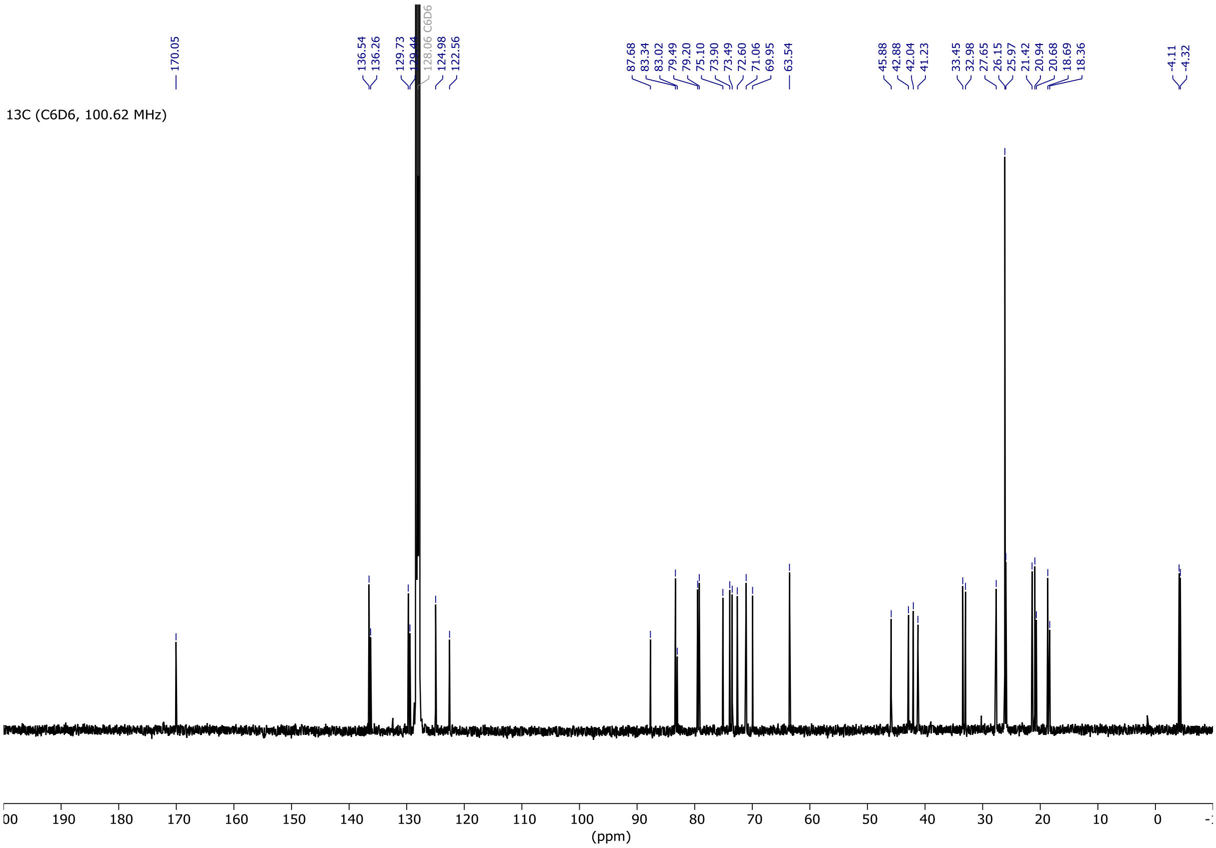
170.05
136.54 136.26 129.73 129.44 128.06 C6D6 124.98 122.56
87.68 83.34 83.02 79.49 79.20 75.10 73.90 73.49 72.60 71.06 69.95 63.54
45.88 42.88 42.04 41.23 33.45 32.98 27.65 26.15 25.97 21.42 20.94 20.68 18.69 18.36 -4.11 -4.32
13C (C6D6, 100.62 MHz)
00 190 180 170 160 150 140 130 120 110 100 90 80 70 60 50 40 30 20 10 0 -1
(ppm)

## Compound 24

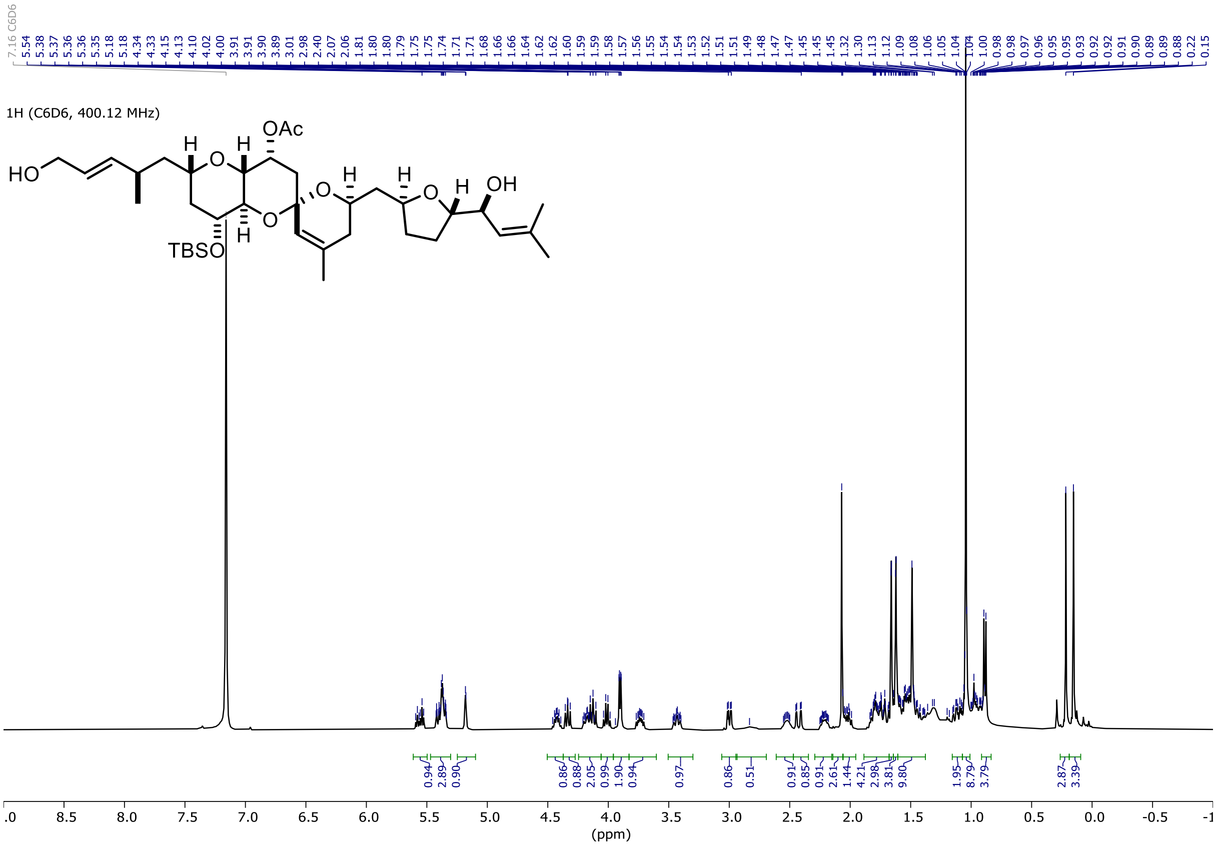
7.16 C6D6
5.54 5.38 5.37 5.36 5.36 5.35 5.18 5.18 4.34 4.33 4.15 4.13 4.10 4.02 4.00 3.91 3.91 3.90 3.89 3.01 2.98 2.40 2.07 2.06 1.81 1.80 1.80 1.79 1.75 1.75 1.74 1.71 1.71 1.68 1.66 1.66 1.64 1.62 1.60 1.59 1.59 1.58 1.57 1.56 1.55 1.54 1.54 1.53 1.52 1.51 1.51 1.49 1.48 1.47 1.47 1.45 1.45 1.45 1.32 1.30 1.13 1.12 1.09 1.08 1.06 1.05 1.04 1.04 1.00 0.98 0.98 0.97 0.96 0.95 0.95 0.93 0.92 0.92 0.91 0.90 0.89 0.88 0.22 0.15
1H (C6D6, 400.12 MHz)
HO
H
H
OAc
O
O
H
H
O
H
OH
TBSO
H
O
0.94 2.89 0.90 0.86 0.88 2.05 0.99 1.90 0.94 0.97 0.86 0.51 0.91 0.85 0.91 2.61 1.44 4.21 2.98 3.81 9.80 1.95 8.79 3.79 2.87 3.39
(ppm)

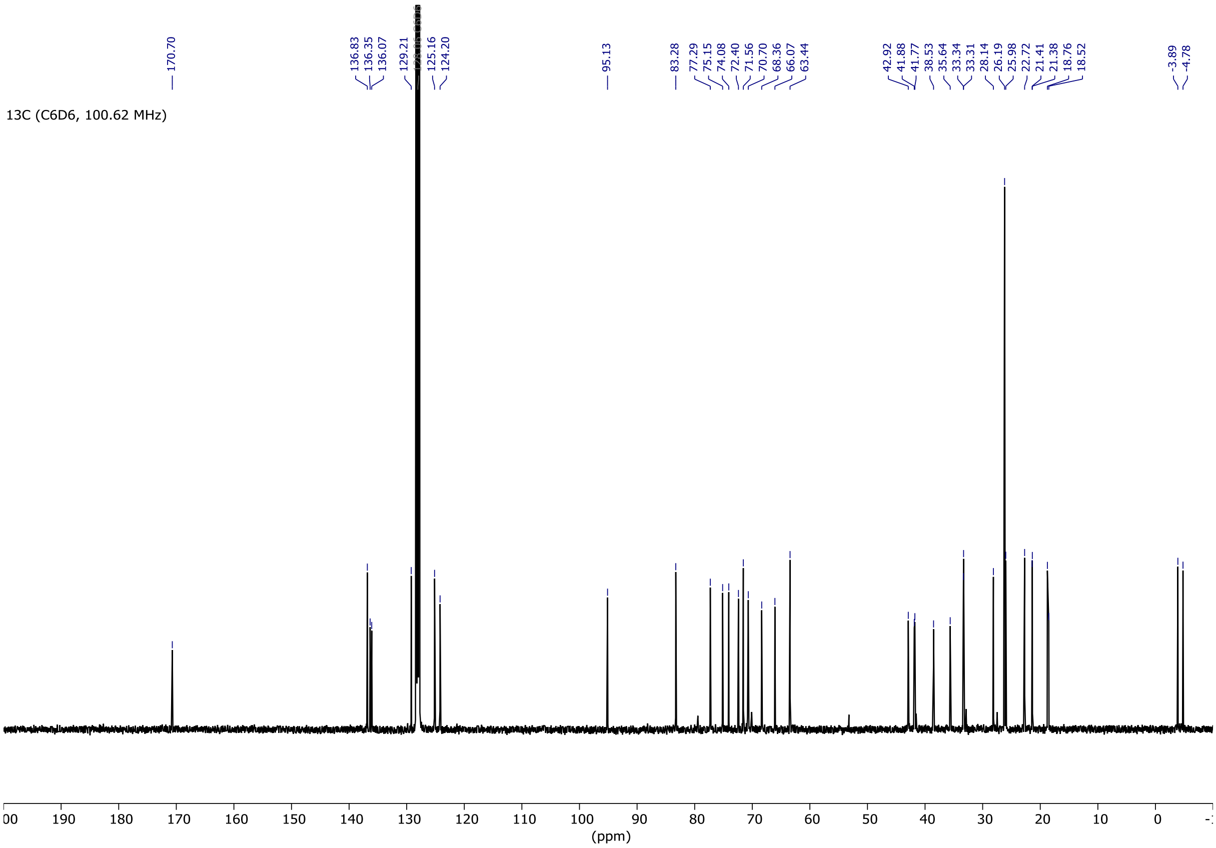
170.70 136.83 136.35 136.07 129.21 125.16 124.20 95.13 83.28 77.29 75.15 74.08 72.40 71.56 70.70 68.36 66.07 63.44 42.92 41.88 41.77 38.53 35.64 33.34 33.31 28.14 26.19 25.98 22.72 21.41 21.38 18.76 18.52 -3.89 -4.78
13C (C6D6, 100.62 MHz)
(ppm)

## Compound 25

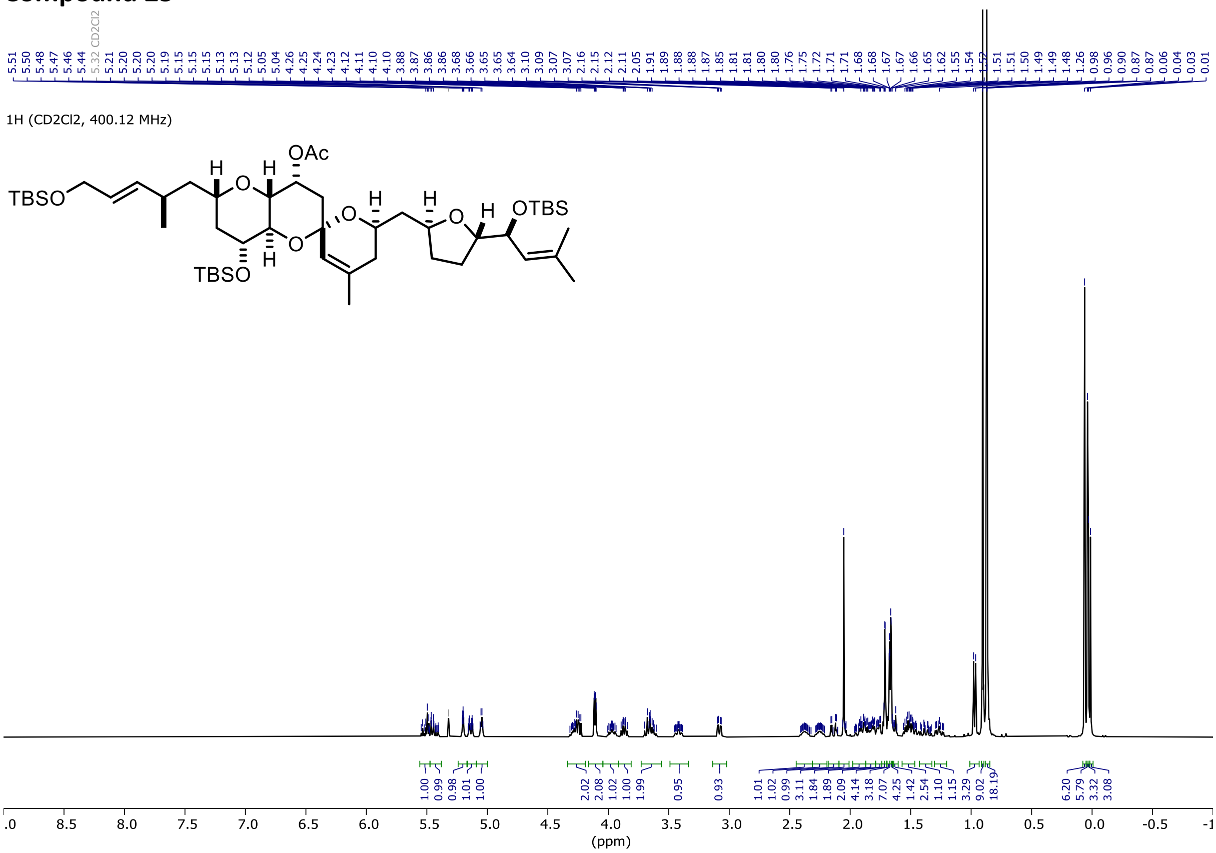
5.51 5.50 5.48 5.47 5.46 5.44 5.32 CD2Cl2 5.21 5.20 5.20 5.20 5.19 5.15 5.15 5.15 5.13 5.13 5.12 5.05 5.04 4.26 4.25 4.24 4.23 4.12 4.11 4.10 4.10 3.88 3.87 3.86 3.86 3.68 3.66 3.65 3.65 3.64 3.10 3.09 3.07 3.07 2.16 2.15 2.12 2.11 2.05 1.91 1.89 1.88 1.88 1.87 1.85 1.81 1.81 1.80 1.80 1.76 1.75 1.72 1.71 1.71 1.68 1.68 1.67 1.67 1.66 1.65 1.62 1.55 1.54 1.52 1.51 1.51 1.50 1.49 1.49 1.48 1.26 0.98 0.96 0.90 0.87 0.87 0.06 0.04 0.03 0.01
1H (CD2Cl2, 400.12 MHz)
OAc
H
H
O
TBSO
O
H
H
O
H
OTBS
TBSO
H
O
1.00 0.99 0.98 1.01 1.00 2.02 2.08 1.02 1.00 1.99 0.95 0.93 1.01 1.02 0.99 3.11 1.84 1.89 2.09 4.14 3.18 7.07 4.25 1.42 2.54 1.10 1.15 3.29 9.02 18.19 6.20 5.79 3.32 3.08
.0 8.5 8.0 7.5 7.0 6.5 6.0 5.5 5.0 4.5 4.0 3.5 3.0 2.5 2.0 1.5 1.0 0.5 0.0 -0.5 -1
(ppm)

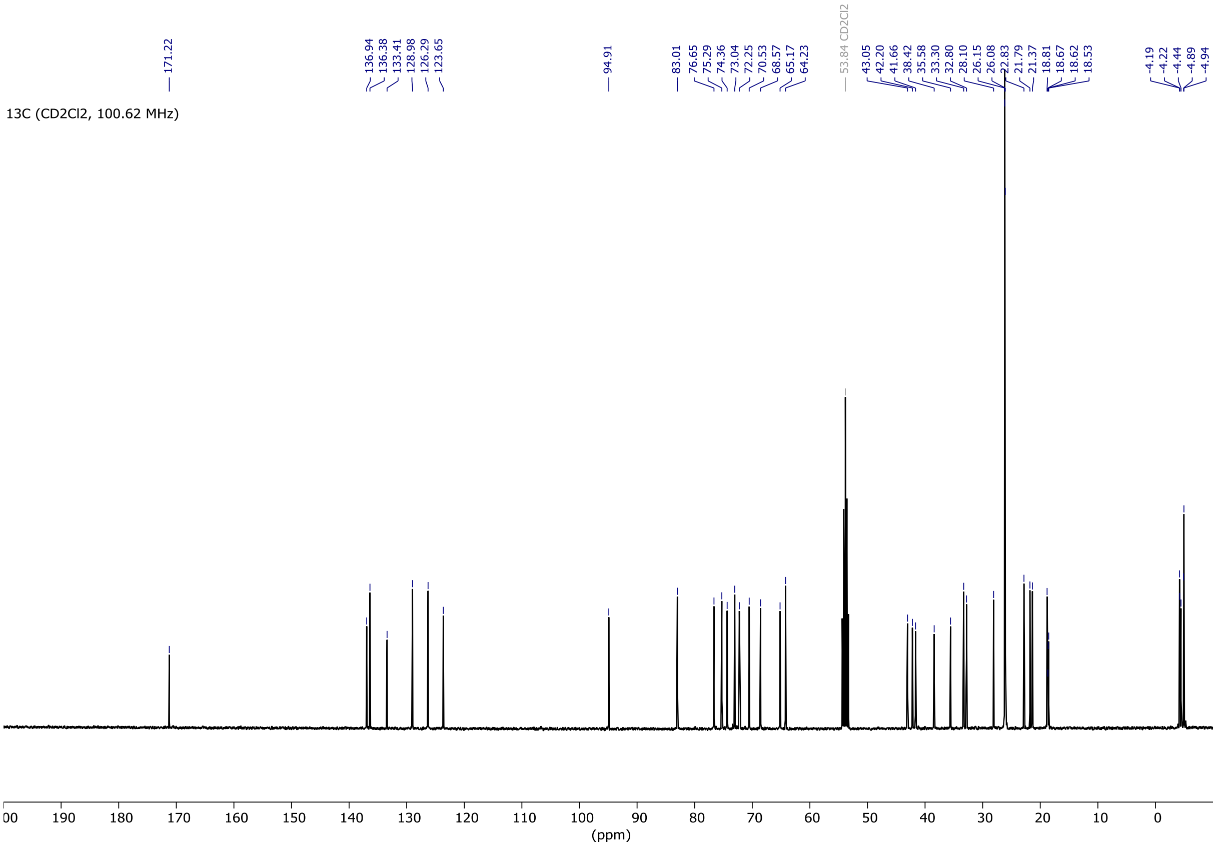
171.22 136.94 136.38 133.41 128.98 126.29 123.65 94.91 83.01 76.65 75.29 74.36 73.04 72.25 70.53 68.57 65.17 64.23 53.84 CD2Cl2 43.05 42.20 41.66 38.42 35.58 33.30 32.80 28.10 26.15 26.08 22.83 21.79 21.37 18.81 18.67 18.62 18.53 -4.19 -4.22 -4.44 -4.89 -4.94
13C (CD2Cl2, 100.62 MHz)
00 190 180 170 160 150 140 130 120 110 100 90 80 70 60 50 40 30 20 10 0
(ppm)

## REFERENCES


1. Arendt, K. M.; Doyle, A. G. Dialkyl Ether Formation by Nickel-Catalyzed Cross-Coupling of Acetals and Aryl Iodides, *Angew. Chem. Int. Ed*. **2015**, *54*, 9876–9880.
2. Ramidi, G. R.; Yadav, J. S.; Mohapatra, D. K. Stereoselective synthesis of the C3-C15 fragment of callyspongiolide, *Tetrahedron Lett*. **2018**, *59*, 3579–3582.
3. Zachmann, R. J.; Yahata, K.; Holzheimer, M.; Jarret, M.; Wirtz, C.; Fürstner, A. Total Syntheses of Nominal and Actual Prorocentin, *J. Am. Chem. Soc*. **2023**, *145*, 2584−2595.
4. Nieto-Oberhuber, C.; López, S.; Echavarren, A. M., Intramolecular [4 + 2] Cycloadditions of 1,3-Enynes or Arylalkynes with Alkenes with Highly Reactive Cationic Phosphine Au(I) Complexes. *J. Am. Chem. Soc.* **2005,** *127* (17), 6178-6179.
